# Killer whale (*Orcinus orca*) interactions with blue-eye trevalla (*Hyperoglyphe antarctica*) longline fisheries

**DOI:** 10.7717/peerj.5306

**Published:** 2018-08-08

**Authors:** Paul Tixier, Mary-Anne Lea, Mark A. Hindell, Christophe Guinet, Nicolas Gasco, Guy Duhamel, John P.Y. Arnould

**Affiliations:** 1 School of Life and Environmental Sciences (Burwood Campus), Deakin University, Geelong, Victoria, Australia; 2 Ecology and Biodiversity Centre, Institute for Marine and Antarctic Studies, University of Tasmania, Hobart, Tasmania, Australia; 3 Centre d’Etudes Biologiques de Chizé (CEBC), UMR 7372 Université de La Rochelle—CNRS, Villiers-en-Bois, France; 4 Département Adaptations du vivant, UMR BOREA, Museum national d’Histoire naturelle, Paris, France

**Keywords:** Fisheries, Killer whale, Fisheries interaction, *Orcinus orca*, Blue-eye trevalla, Hyperoglyphe antarctica, Depredation, Longline fisheries

## Abstract

Over the past five decades, marine mammal interactions with fisheries have become a major human-wildlife conflict globally. The emergence of longline fishing is concomitant with the development of depredation-type interactions i.e., marine mammals feeding on fish caught on hooks. The killer whale (*Orcinus orca*) is one of the species most involved in depredation on longline fisheries. The issue was first reported in high latitudes but, with increasing expansion of this fishing method, other fisheries have begun to experience interactions. The present study investigated killer whale interactions with two geographically isolated blue-eye trevalla (*Hyperoglyphe antarctica*) fisheries operating in temperate waters off Amsterdam/St. Paul Islands (Indian Ocean) and south-eastern Australia. These two fisheries differ in the fishing technique used (vertical *vs.* demersal longlines), effort, catch, fleet size and fishing area size. Using 7-year (2010–16) long fishing and observation datasets, this study estimated the levels of killer whale interactions and examined the influence of spatio-temporal and operational variables on the probability of vessels to experience interactions. Killer whales interactions occurred during 58.4% and 21.2% of all fishing days, and over 94% and 47.4% of the fishing area for both fisheries, respectively. In south-eastern Australia, the probability of occurrence of killer whale interactions during fishing days varied seasonally with a decrease in spring, increased with the daily fishing effort and decreased with the distance travelled by the vessel between fishing days. In Amsterdam/St. Paul, this probability was only influenced by latitude, with an increase in the southern part of the area. Together, these findings document two previously unreported cases of high killer whale depredation, and provide insights on ways to avoid the issue. The study also emphasizes the need to further examine the local characteristics of fisheries and the ecology of local depredating killer whale populations in as important drivers of depredation.

## Introduction

Over the past five decades, marine mammal-fisheries interactions have become a major issue globally (Northridge, 1991; Read, 2008). Depredation-type interactions occur when marine mammals partially or completely consume bait and/or fish caught on fishing gear (Gilman et al., 2007; Hamer, Childerhouse & Gales, 2012). The issue has been reported in many commercial and artisanal fisheries, and involves a broad range of marine mammal species and fishing techniques (Read, 2008). Longline fishing, which has developed over the past 60 years, is the technique currently experiencing the highest levels of such depredation at all latitudes of both hemispheres, primarily by odontocetes (toothed whales) (Hamer, Childerhouse & Gales, 2012; Werner et al., 2015).

Odontocete depredation on longline fisheries can have dire socio-economic and ecological consequences (Hamer, Childerhouse & Gales, 2012; Werner et al., 2015). Firstly, the issue often results in substantial financial losses to the fishers. The amount of fish or bait odontocetes removed from longlines imposes increased fishing time and displacements to achieve quotas and/or to implement strategies of avoidance (Peterson et al., 2014; Thode et al., 2016). Secondly, the amount of depredated fish is often inaccurately assessed or unaccounted when setting catch limits, resulting in biased fish stock assessments (Peterson et al., 2013; Peterson & Hanselman, 2017; Hanselman, Pyper & Peterson, 2018). Finally, depredation may negatively impact the conservation of depredating odontocete populations, primarily through death/injuries from interactions with fishing gear and lethal response from fishers (Matkin & Saulitis, 1994; Gilman et al., 2007; Hamer, Childerhouse & Gales, 2012; Tixier et al., 2017).

The killer whale (*Orcinus orca*) is one of the odontocete species most involved in depredation on longline fisheries. The species has been reported primarily depredating on demersal longlines at high latitudes of both hemispheres (Matkin & Saulitis, 1994; Yano & Dahlheim, 1995; Kock, Purves & Duhamel, 2006), and on pelagic longlines in temperate regions (Rosa & Secchi, 2007; Passadore, Domingo & Secchi, 2015). Previous studies have suggested that killer whales, which often depredate in groups and can be highly effective in removing large amounts of fish from longlines, have substantially greater impacts on local fishing industry and management than any other depredating species (Hucke-Gaete, Moreno & Arata, 2004; Roche et al., 2007; Clark & Agnew, 2010; Tixier et al., 2010; Peterson et al., 2013; Gasco et al., 2015). In some cases, high depredation levels have been reported to significantly impact the demography and, thus, the conservation of local populations of depredating killer whales, either negatively or positively (Tixier et al., 2015; Esteban et al., 2016a; Esteban et al., 2016b; Tixier et al., 2017).

Most of the research effort on killer whale depredation has focused on high value commercial fisheries for which the issue has been reported, such as the Patagonian toothfish (*Dissostichus eleginoides*) longline fisheries in the Southern Ocean, the sablefish (*Anoplopoma fimbria*) longline fisheries in Alaska or the swordfish (*Xiphias gladius*) and tuna (*Thunnus spp.*) longline fisheries in temperate waters of the Atlantic Ocean. Extensive studies have been conducted to assess the levels of depredation (Secchi & Vaske, 1998; Rosa & Secchi, 2007; Peterson et al., 2013; Gasco et al., 2015; Passadore, Domingo & Secchi, 2015), to better understand the ecology of local whale populations (Fearnbach et al., 2014; Guinet et al., 2014), and to identify ways to minimize the issue through strategies of avoidance or technological solutions (Moreno et al., 2008; Tixier et al., 2014a; Tixier et al., 2014b; Wild et al., 2017). Results from these studies have been used to improve monitoring and management of local fish stocks and to prioritize actions for the management of killer whale populations (Tixier et al., 2015; Peterson & Hanselman, 2017; Tixier et al., 2017). However, other longline fisheries targeting fish species of moderate commercial value are facing depredation by killer whales, which has received little research attention and, in some cases, may jeopardize the viability and the sustainability of the local fishing activity.

The blue-eye trevalla (*Hyperoglyphe antarctica*) longline fisheries operate in the temperate waters of the southern hemisphere. Such fisheries occur at the edge of continental shelves and around islands/seamounts in South America, South Atlantic Ocean, South Indian Ocean and around south-eastern Australia and New Zealand (Bensch et al., 2008). Among these areas, the Exclusive Economic Zones (EEZs) of New Zealand and Australia host the most economically important commercial fishing activity for blue-eye trevalla. In south-eastern Australia (130–152°E; 33–45°S—hereafter the “SE Australia” fishery), the species has been historically caught through commercial bottom trawling, drop-lining and auto-lining since 1986 at depth ranging from 200 to 600 m (Haddon, 2012; Haddon, 2015). Demersal auto-lining has become the predominant fishing technique in 2004, and since 2010 between five and six vessels are licensed annually to use this fishing technique in SE Australia with a blue eye trevalla catch ranging from 200 to 300 t per year (Haddon, 2015; Helidoniotis et al., 2017). However, there have been five vessels significantly contributing to the total catch (>10 tonnes per year) from 2010 to 2012, and four vessels contributing to that level since 2013 (Haddon, 2012; Haddon, 2015; Helidoniotis et al., 2017).

In the South Indian Ocean, blue eye trevalla is caught in the French EEZ of Amsterdam and St Paul Islands (77–78°E; 37–40°S—hereafter the “Amsterdam/St. Paul” fishery) as a secondary species by one licenced commercial fishing vessel primarily targeting the Saint Paul rock lobster (*Jasus paulensis*). In this area, blue eye trevalla has been targeted as a commercial fish since the early 2000s using bottom longlining until 2009, and then exclusively using vertical longlining since 2010 as part of a multispecies finfish longline fishery, with an annual catch ranging from 10 to 20 t (Pruvost et al., 2015). Killer whale depredation has been reported as occurring in both the SE Australia and Amsterdam/St. Paul fisheries (Shaughnessy et al., 2003; Pease, 2012; C Guinet, pers. comm., 2018). However, estimates of the extent to which killer whales interact with fishing operation are limited such that the economic and ecological impacts of depredation are unknown. Such knowledge is crucial if mitigation measures are to be developed (Guinet et al., 2014).

The aims of the present study, therefore, were to: (1) estimate the levels of killer whale depredation in two local blue-eye trevalla fisheries; (2) assess their spatial and temporal variation and (3) provide preliminary insights on ways to minimize these interactions.

## Material & Methods

**Figure 1:**
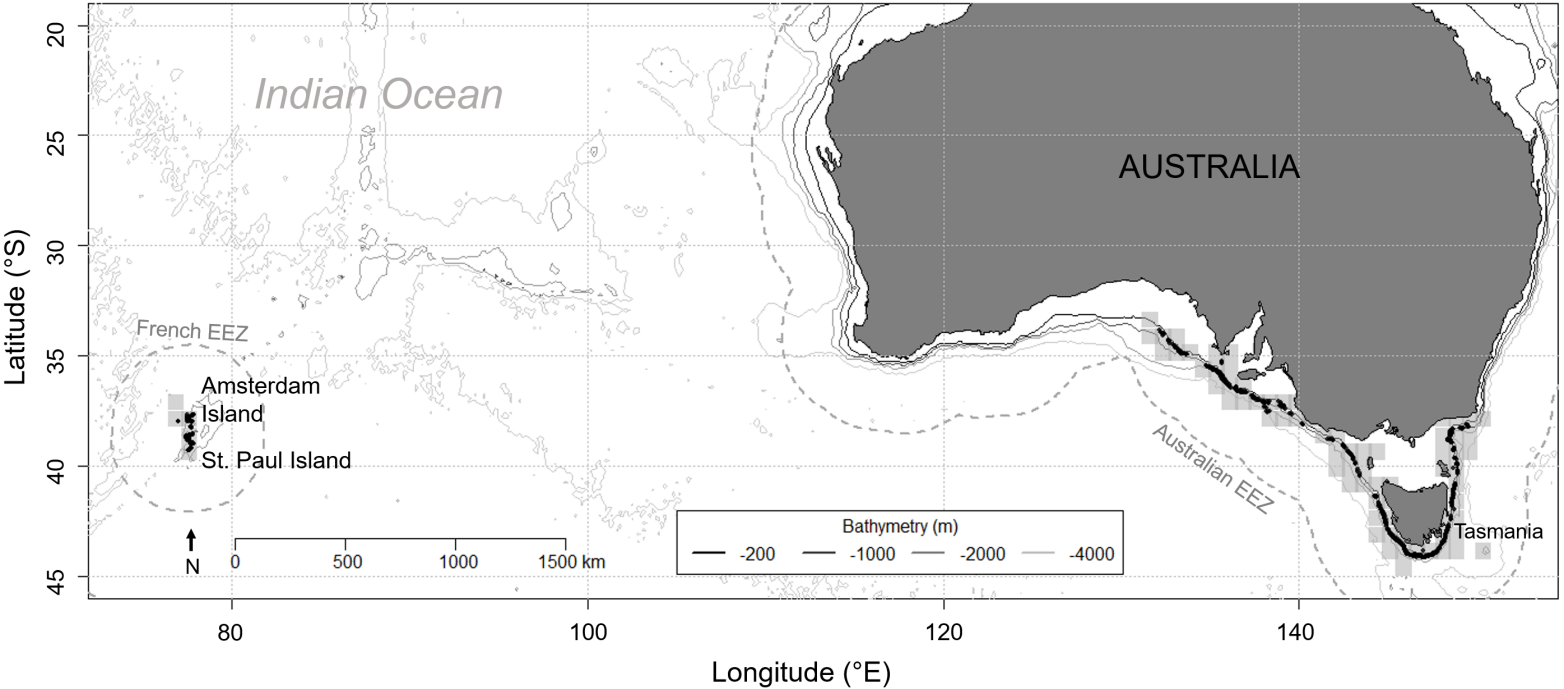
Location of the two study areas: Amsterdam and Saint Paul Islands within the French EEZ and South East Australia as part of the Australian EEZ. Grey shaded squares depict the areas where longline fishing for blue eye trevalla occurred and black dots are the locations of longline sets for which killer whale interaction was recorded during hauling, from 2010 to 2016 in the Australian EEZ and from 2015 to 2016 in the French EEZ. The two EEZs are delineated by a grey dashed line.

Fishing and observation data were collected in SE Australia and in Amsterdam/St. Paul from 7 January 2010 to 31 December 2016 (Fig. 1). In SE Australia, data were retrieved from log books recorded from one of the fishing vessels catching more than 10 tonnes per year (representing 20%–25% of the contributing vessels of the fleet depending on year) by two separate captains. In Amsterdam/St. Paul, data were collected by fishery observers from the only vessel licensed to operate in this area, and retrieved from the PECHEKER database (Martin & Pruvost, 2007). In both areas, 100% of the fishing operations by monitored vessels, here defined as either setting or hauling of a longline set (i.e., a main line with baited hooks), were monitored. A total of 5,451 demersal longlines were monitored in SE Australia, with an average of 779 ±  52 SE sets per year (*n* = 7 years) and 5 ± 0.1 SE sets per fishing day (*n* = 1,095 days). In Amsterdam/St. Paul, 3,354 vertical longlines were monitored, with an average of 479 ± 223 SE sets per year (*n* = 7 years) and 14 ± 1 SE sets per fishing day (*n* = 242 days). Demersal longlines in SE Australia averaged 2,918 ± 18 SE hooks per set (*n* = 5,451 sets) and vertical longlines in Amsterdam/St. Paul averaged 136 ± 3 SE hooks per set (*n* = 3,354 sets).

For each longline set, data included the date of hauling, and the occurrence of killer whale depredation during hauling. This occurrence was recorded as three distinct states: “presence”, “absence” and “unknown”. If weather/light conditions were suitable for surface observation (visibility > 2 miles) and fishing effort data was available: whales visually confirmed depredating from behavioural cues (repeated long dives towards the line being hauled, surrounded by seabirds when surfacing, slicks of fish oil visible at the surface of the water and/or chunks of fish observed in the mouth of whales) was classified as “presence”; and no whales sighted from the vessel or, if sighted, whales were in transit with no indicators of depredation (see above), was classified as “absence”. If weather/light conditions were not suitable for observation and/or no fishing effort data were available, the occurrence of depredation for that line was recorded as “unknown”. The GPS coordinates of the location at which longlines were set were systematically recorded in SE Australia during the whole study period, but only for years 2015 and 2016 in Amsterdam/St. Paul. Coordinates were converted negative decimal degrees for latitude and positive decimal degrees for longitude.

The level of killer whale–vessel interaction was measured as two indices: the proportion of fishing days (noted “*Pr(days)*”); and the proportion of the fishing area (noted “*Pr(area)*”). *Pr(days)* was calculated as the number of fishing days (i.e., days when a minimum of one longline set was hauled) during which at least one set was hauled in presence of interacting killer whales out of all fishing days. *Pr(area)* was calculated as the number of spatial cells (defined over a 0.1° latitude × 0.1° longitude grid) inside which at least one set was hauled by one vessel in presence of interacting killer whales out of all cells inside which longline sets were hauled by this vessel. To limit bias due to uncertainty in the occurrence of killer whale interactions, for both *Pr(days)* and *Pr(area)*, days or cells during/in which none of the sets were hauled in presence of killer whales but at least one set was hauled with an “unknown” record were removed from calculations.

Generalized linear models (GLM, *glm* in R package *stats*) were used to examine the spatio-temporal variations of killer whale interactions with blue-eye trevalla fishing vessels in Amsterdam/St. Paul and SE Australia. The response variable was the occurrence of interactions during fishing days. The two states of this variable, either “0” or “1” were retrieved from the records of presence (1) or absence (0) of killer whales during hauling of longline sets as follows: fishing days during which killer whales were observed interacting with at least one longline set were assigned a (1) and fishing days during which longline sets were all hauled in the confirmed absence of killer whales were assigned a (0). Days during which at least one set was hauled with an “unknown” state in regards to the presence of killer whales were excluded from the analysis. Models were fitted with a binomial distribution and a log link function.

As shown in previous studies, the data on presence/absence of killer whale interaction were likely to be autocorrelated in time and in space over consecutive fishing operations (Tixier et al., 2014a; Tixier et al., 2014b; Janc et al., 2018). To account for such autocorrelation, we included the presence/absence records of killer whales during the previous fishing day and the distance travelled by vessels between this previous day and the next, as an interaction structural term in the null model (Data S1). The data were ordered by chronological order of fishing days, which were consecutive since vessels fished every day during trips. The distance travelled was calculated as the Euclidean distance using the mean latitude and longitude at which vessels operated during one day and the previous one using the *spDistsN1* in R package *sp*. Autocorrelation was tested on models including and excluding this structural term using the *acf* function in R package *stats* (Venables & Ripley, 2013).

Time variables were included in models as two predictors: year (continuous) and season (categorical), to respectively evaluate whether a trend existed over the study period, and to explore seasonality in the interaction patterns. Seasons were defined according to the meteorological definition: *winter* (1 June–31 August); *spring* (1 September–30 November); *summer* (1 December–28 February); and *autumn* (1 March–31 May). The mean latitude and longitude of fishing operations, calculated for each fishing day using locations of all sets hauled during that day, were tested in models as two continuous spatial predictors (Table 1). The mean depth at which longlines hauled during a given day were set, which ranged from 56 to 696 m in Amsterdam/St. Paul, and from 133 to 896 m in SE Australia (Table 1 and Data S1) was also added to models.

**Table 1:** Predictors considered in the Generalised Linear Models fitted to the occurrence of killer whale interactions during fishing days. Description of the type of term (continuous or categorical), the unit, and the range of values or levels taken is provided for the two studied fisheries: Amsterdam/St. Paul and in SE Australia.

			Amsterdam/St. Paul	SE Australia
Term	Type	Unit	Range/levels	Range/levels
Year	continuous	calendar year	[2010 : 2016]	[2010 : 2016]
Season	categorical	meteorological seasons	3 summer/autumn/spring	4 summer/autumn/winter/spring
Latitude	continuous	decimal^∘^ South	[37.7 : 39.3]	[33.6 : 44.2]
Longitude	continuous	decimal^∘^ East	[76.4 : 77.9]	[131.7 : 150.4]
Depth	continuous	meters	[56 : 697]	[133 : 893]
Effort per day	continuous	hooks	[400 : 7,875]	[1,150 : 29,900]
Size of the fished area per day	continuous	spatial 0.1^∘^ × 0.1^∘^ cells	[1 : 5]	[1 : 8]
Distance from previous fishing day within trips	continuous	kilometers	[0 : 146]	[0.7 : 680]
Data collection	categorical	observers/captains	4 observers	2 captains

Two operational variables were tested as continuous fixed effects: the size of the area fished by a vessel during a fishing trip, and the total fishing effort provided a vessel during a fishing day. The size of the fished area was calculated as the total number of 0.1° × 0.1° cells in which at least one set was hauled during a fishing day as an index of spatial spread of effort. The fishing effort was calculated as the total number of hooks hauled during a fishing day. Both operational variables were tested under the assumption that increased fishing effort and spatial spread of effort would increase the probability for killer whales to detect and to converge on fishing gear, therefore increasing the probability of interaction (Passadore et al., 2014; Cruz et al., 2016).

Finally, potential variations in the way data were collected in the studied fisheries were accounted for by including the identity of the four different observers in Amsterdam/St. Paul, and the identity of the two different captains that collected the data in SE Australia in the models. All continuous predictors were standardized to facilitate model convergence and models were fitted separately for each of the two study areas. Model selection was conducted using the Akaike Information Criterion (AIC) and a forward stepwise procedure (Burnham & Anderson, 2003) (Data S1). Unless otherwise stated data are presented as mean ±  SE.

## Results

From 2010 to 2016, killer whales interacted with blue-eye trevalla longlines during 142 days in Amsterdam/St. Paul and 232 days in SE Australia, which represented *Pr(days)* = 58.7% and 21.2% of 242 and 1,095 fishing days, respectively.

**Figure 2:**
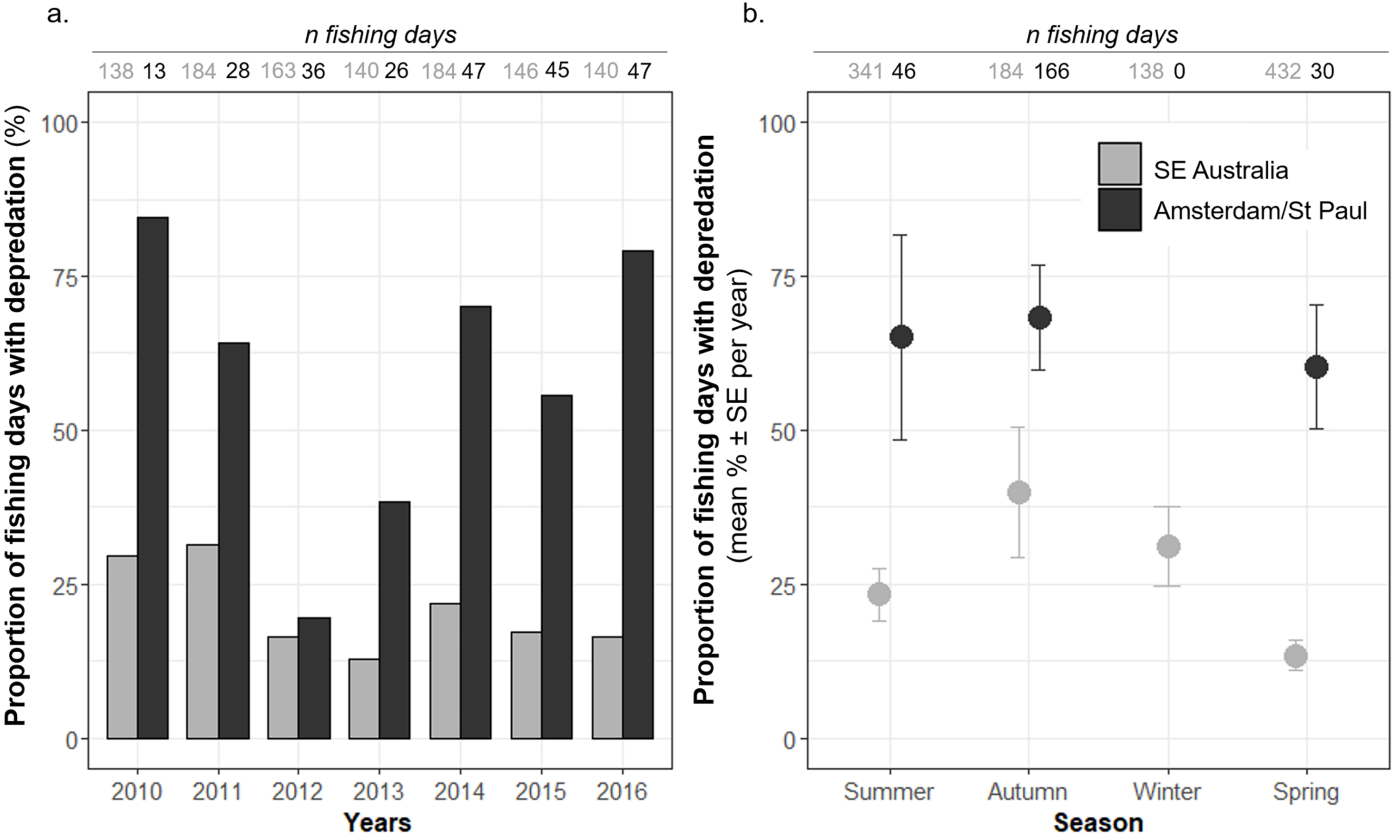
Time variations of the observed proportions of fishing days during which killer whale interaction with the fishing gear occurred out of all fishing days (*Pr(days)*). *Pr(days)* was calculated (A) per year, and (B) per season, from 2010 to 2016, in Amsterdam/St. Paul (black) and in SE Australia (grey).

The most parsimonious GLM fitted to the occurrence of killer whale interactions during fishing days included the latitude and the year as predictors for Amsterdam/St. Paul, and the season, the fishing effort and the captain identity for SE Australia (Table 2 and Data S1). In SE Australia, *Pr(days)* varied from 29.7% in 2010 to 16.4% in 2016, with a maximum of 31.5% reached in 2011 (Fig. 2A) but no trend was statistically detected (Table 2)*. Pr(days)* also varied between years in Amsterdam/St. Paul, with a maximum of 84.6% in 2010 and a minimum of 19.4% in 2012 (Fig. 2A). However, while the year was selected in the final model and model outputs indicated a positive trend, this trend was not significant (*z* = 1.792, *P* = 0.073).

**Table 2:** Summary outputs and parameter estimates of the best GLM-type model fitted on the presence/absence records of killer whale interaction with blue eye trevalla longline sets during fishing days. Continuous predictors were the year, the latitude, the longitude, the depth at which vessels operated during fishing days, as well as the distance travelled from one day to the next and the daily fishing effort. The time of year vessels operated was tested through the Season as a categorical predictor, with the effects of autumn, winter and spring tested against the summer effect. The identity of the person who collected the data, which was a fishery observer in Amsterdam/St. Paul and the captain in SE Australia, was included as a categorical predictor. Outputs are provided for predictors that were included in the final model after a forward stepwise AIC selection, “ns” indicates that the predictor was not selected in the final model, and (−) indicates that the predictor was not tested.

	Amsterdam/St. Paul				SE Australia			
Predictors	Estimate	SE	*z*	*P*	Estimate	SE	*z*	*P*
Intercept	−0.133	0.391	−0.341	0.733	−1.343	0.197	−6.813	<0.001
Killer whale presence (previous day)	1.547	0.516	2.997	0.003	2.519	0.217	11.613	<0.001
Distance travelled (previous day)	0.457	0.585	0.782	0.434	−0.330	0.164	−2.016	0.044
Killer whale presence * distance travelled (previous day)	−0.806	0.627	−1.284	0.199	−1.043	0.322	−3.236	0.001
Year	0.506	0.282	1.792	0.073	ns			
Season: *autumn*	ns				−0.163	0.256	−0.637	0.524
Season: *winter*	–				−0.627	0.391	−1.605	0.108
Season: *spring*	ns				−0.532	0.247	−2.148	0.032
Latitude	0.825	0.327	2.523	0.012	ns			
Longitude	ns				ns			
Depth	ns				ns			
Effort	ns				0.381	0.099	3.840	<0.001
Size of area fished	ns				ns			
Observer	ns				–			
Captain: captain 2	–				−1.212	0.229	−5.287	<0.001

In SE Australia, *Pr(days)* also varied between seasons, with a minimum of 13.4 ± 2.5% per year (*n* = 7 years) in spring, which was significantly lower than in summer (23.3 ± 4.3% per year—*n* = 7 years) as indicated by the model (*z* =  − 2.148, *P* = 0.032—Table 2 and Fig. 1B). The maximum was in autumn with 39.9 ± 10.7% of the fishing days per year (*n* = 7 years). In Amsterdam/St. Paul, the fishing vessel never operated in winter and *Pr(days)* was maximal in autumn with 68.3 ± 8.5 SE% and minimal in spring with 60.4 ± 10.1 SE% per year (*n* = 7 years), but variations were not significant between seasons (Table 2 and Fig. 2B).

**Figure 3:**
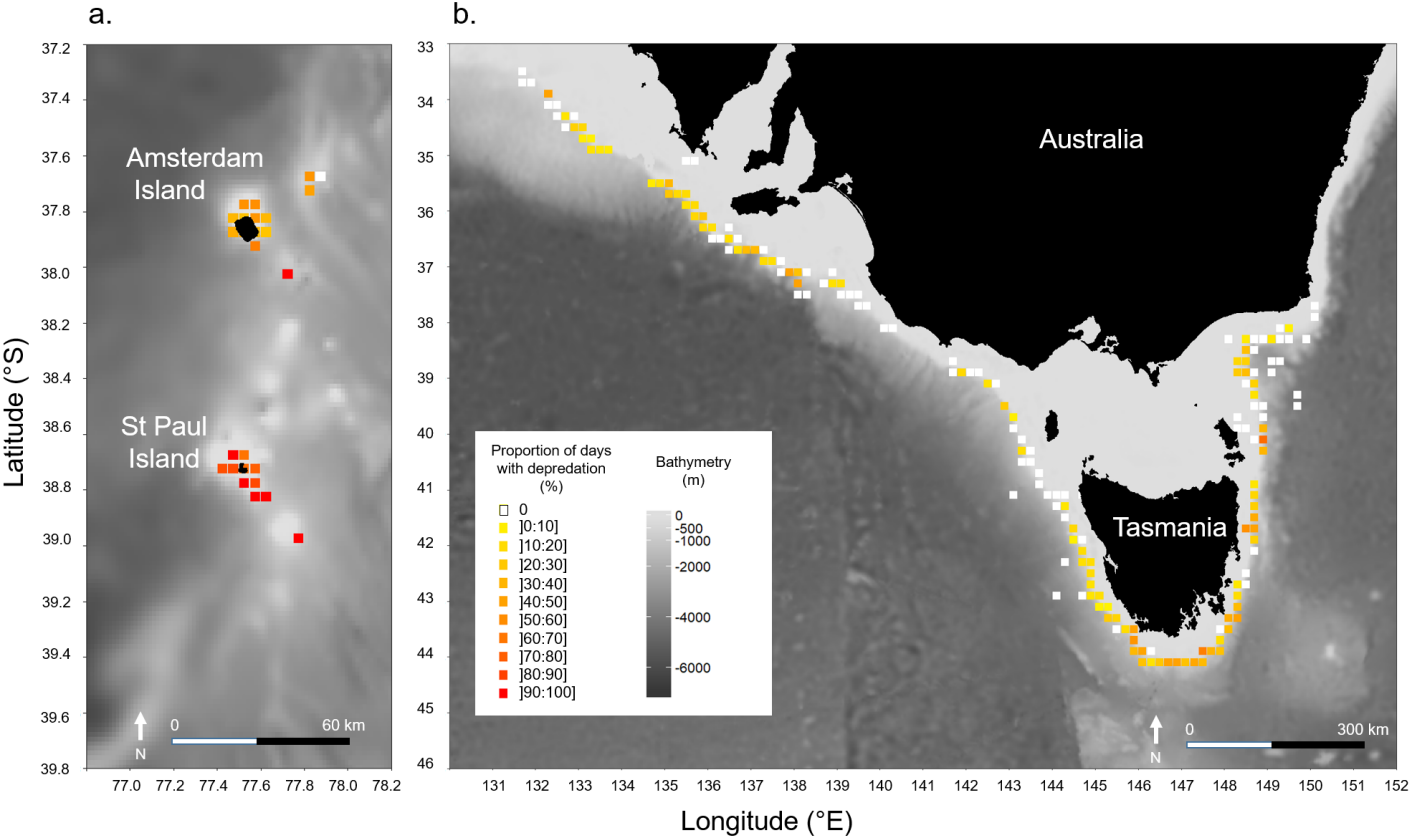
Spatial variations of the observed proportions of fishing days during which killer whale interaction with the fishing gear occurred out of all fishing days (*Pr(days)*). *Pr(days)* was calculated over a 0.1° × 0.1° grid: (A) in Amsterdam/St. Paul from 2015 to 2016, and (B) in SE Australia from 2010 to 2016.

Models indicated that the occurrence of killer whale interaction during days was not significantly influenced by the depth in the areas at which vessels operated in neither of the two fisheries (Table 2). In Amsterdam/St. Paul, for which location data on fishing operations were available for 2015 and 2016 only, latitude had a significant positive effect (*z* = 2.523, *P* = 0.012) suggesting a significant increase in the probability of killer whale interactions as the vessel operated further south within the area (Table 2). This was supported by the spatial visualisation of *Pr(days)* on a 0.1° × 0.1° grid, which showed that *Pr(days)* was systematically >70% when the vessel operated around St. Paul Island (Fig. 3A). In SE Australia, the occurrence of killer whale interactions during fishing days did not vary linearly with either latitude or longitude. However, spatial variations of *Pr(days)* were visible with high values (>30% of fishing days) in the southernmost fishing areas around Tasmania and westernmost fishing areas between 132–139°E (Fig. 3B). Conversely, fishing areas located off north-western Tasmania and off the south-eastern part of the Australian mainland showed low *Pr(days)* values (<10% of fishing days).

**Figure 4:**
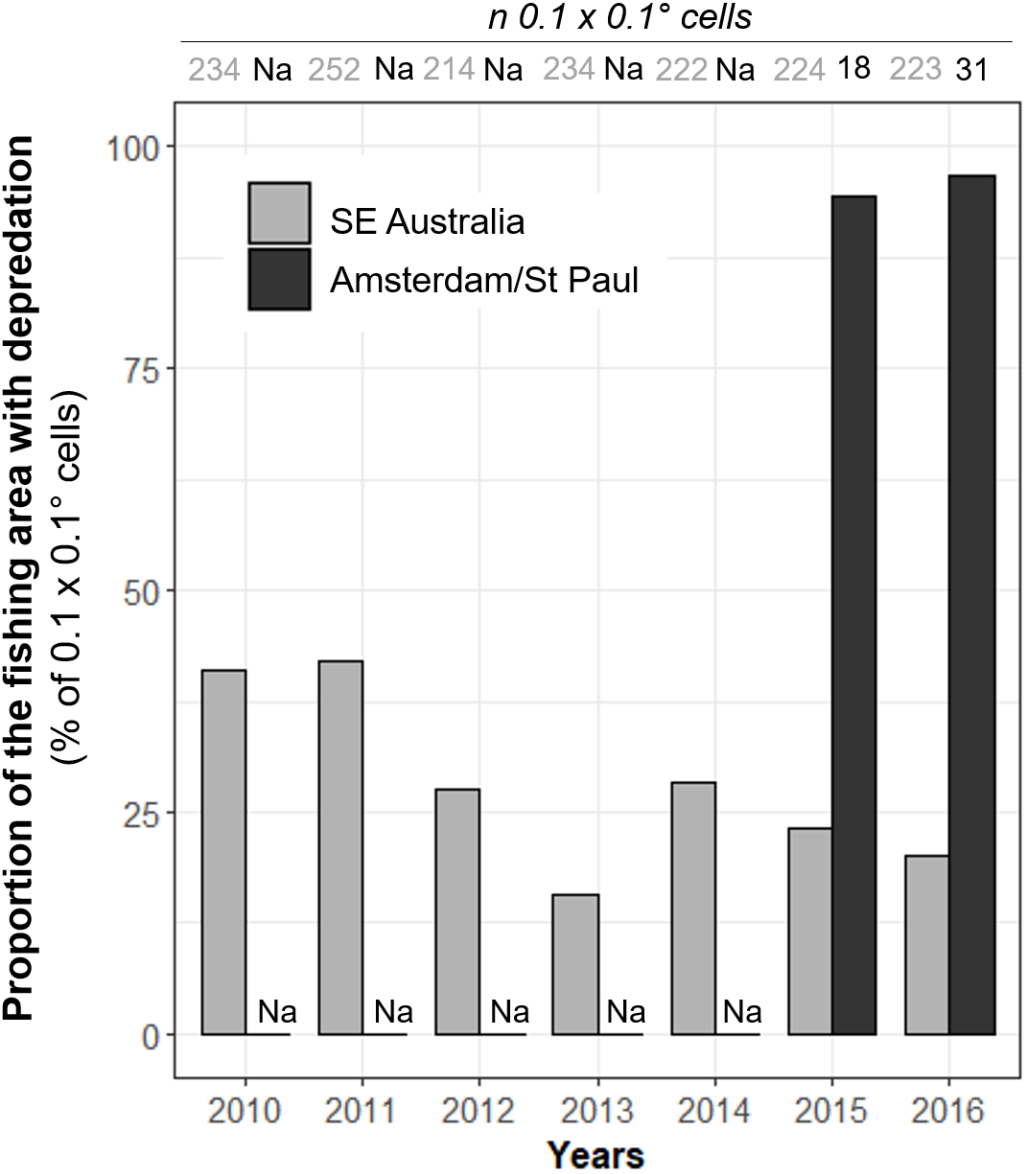
Annual variations of the observed proportions of 0.1° × 0.1° spatial cells within which killer whale interaction with the fishing gear occurred out of all cells in which vessels operated (*Pr(area)*). Annual values of *Pr(area)* were calculated from 2010 to 2016 in SE Australia (grey) and from 2015 to 2016 in Amsterdam/St. Paul (black).

These spatial variations were further emphasized by the proportion of the fishing area where killer whale interaction occurred. Overall, killer whales in SE Australia interacted with vessels in *Pr(area)* = 47.4% of all 0.1° × 0.1° spatial cells in which the vessel operated during 2010–2016, with a maximum of 42.1% in 2011 and a minimum of 15.8% in 2013 (Fig. 4). *Pr(area)* was substantially higher in Amsterdam/St. Paul, with 94.4% and 96.8% of cells in 2015 and 2016, respectively (Fig. 4.).

**Figure 5:**
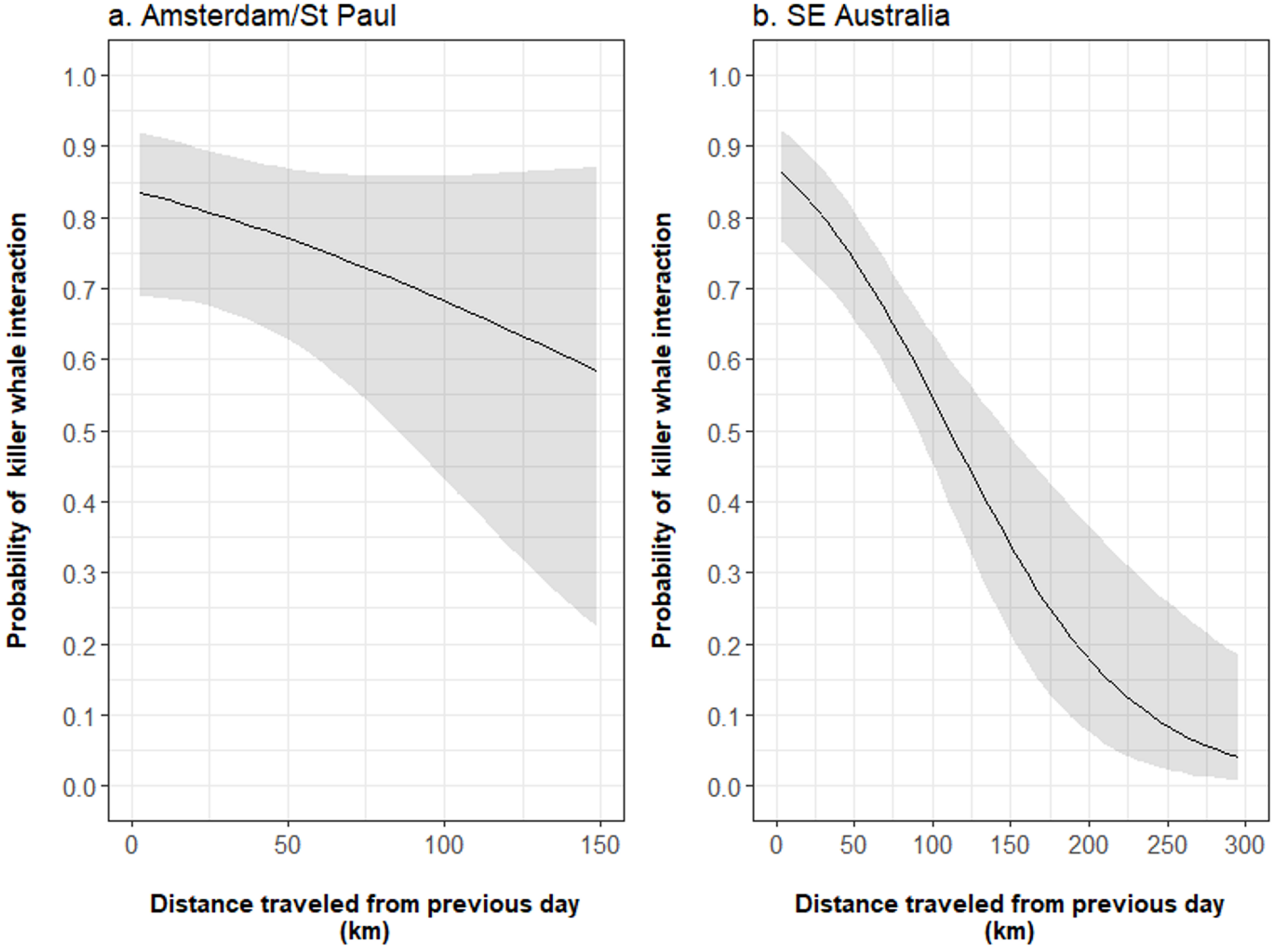
Influence of the distance travelled by vessels from one fishing day during which killer whale interaction occurred to the next on the probability of killer whale interaction during the next day. The mean probability (black line) and 95% confidence intervals (grey shade) were estimated from the output parameters of the final Generalised Linear Models fitted to the occurrence of killer whale interaction during fishing days in (A) Amsterdam/St. Paul and (B) SE Australia.

In both fisheries, the structural interaction term of the occurrence of killer whales during the previous day and the distance travelled between this day and the next reduced the autocorrelation in the data to insignificant levels (Data S1). This term had a significant negative effect on the occurrence of killer whale interaction during a given fishing day and a large slope in SE Australia (−1.043 ± 0.322, *z* =  − 3.236, *P* = 0.001) but was not significant in Amsterdam/St. Paul (−0.806 ±  0.627, *z* =  − 1.284, *P* = 0.199; Table 2). From the model outputs, when interactions with killer whales occurred during a given day in Amsterdam/St. Paul, the probability of killer whale interactions during the subsequent day decreased from 0.84 [95% CI [0.69–0.92]], when the vessel travelled 3 km, to 0.68 [95% CI [0.43–0.86]], when it travelled 100 km between these two days (Fig. 5A). In SE Australia, this probability decreased from 0.86 [95% CI [0.77–0.92]] when the vessel travelled 3 km, to 0.53 [95% CI [0.44–0.63]] when it travelled 100 km (Fig. 5B). While the size of the area fished during fishing days was not selected in final models for either of the two fisheries, the fishing effort during fishing days had a positive significant effect on the occurrence of killer whale interactions in SE Australia (*z* = 3.840, *P* < 0.001). From the model outputs, the estimated probability of killer whale interaction during fishing days increased from 0.18 [95% CI [0.12–0.27]] for 2,000 hooks per day to 0.39 [95% CI [0.34–0.46]] for 20,000 hooks.

Finally, a captain effect was detected in SE Australia with a significant difference in the occurrence of killer whale interactions between the two captains that operated the studied vessel (Table 2). No effect of the observer identity was detected in Amsterdam/St. Paul.

## Discussion

Whereas killer whales have primarily been reported interacting with demersal longline fisheries in latitudes >40° in both hemispheres, depredation by the species in warm and temperate waters has primarily been previously reported on pelagic longlines and artisanal drop-lines, targeting mainly tuna and/or swordfish (Rosa & Secchi, 2007; Passadore, Domingo & Secchi, 2015; Esteban et al., 2016a; Esteban et al., 2016b). The present study documented the depredation by killer whales on demersal longline fisheries operating in two previously unassessed areas located between 30 and 45° South, the lowest latitude range of interactions with these fisheries.

Killer whale—blue eye trevalla fisheries interactions in Amsterdam/St. Paul and SE Australia occurred consistently over multiple years, as has been observed in the blue-eye trevalla demersal longline fishery in New Zealand (Visser, 2000). The frequencies of killer whale–fisheries interactions reported in the present study (16–85% of the fishing days per year) are high and comparable to those reported in areas of severe depredation by the species such as the Crozet Islands (on average 43.5% of the longline sets, Tixier et al., 2016). The interactions in the blue-eye trevalla fisheries, therefore, are likely to result in large amounts of fish being removed from longlines with potentially significant impacts on the local fishing industry, fish stocks and the killer whale populations involved (Peterson et al., 2014; Tixier et al., 2015; Peterson & Hanselman, 2017; Tixier et al., 2017; Hanselman, Pyper & Peterson, 2018). In Amsterdam/St. Paul, field observations suggest that killer whales exclusively remove blue-eye trevallas from hooks and disregard the other species (e.g., Hapuku *Polyprion oxygeneios* or striped trumpeter *Latris lineata*) caught on longline sets (G Duhamel, pers. comm., 2018). In SE Australia, preliminary studies reported approximate decreases of 20–80% in blue-eye trevalla catch rates when killer whales interacted with the fishing gear (AFMA, 2005; Pease, 2012). However, the impacts of killer whale depredation in these fisheries have yet to be properly assessed. Consequently, priority research efforts should be directed towards estimating the amount of blue-eye trevalla removed by killer whales (Gasco et al., 2015; Hanselman, Pyper & Peterson, 2018).

Depredation interactions were greater in the Amsterdam/St. Paul than in SE Australia. This difference could be due to combination of factors such as: (i) the size of the fishing area, (ii) the fleet size, (iii) the density of vessels operating on fishing areas, and (iv) the number of depredating killer whales. The size of the fishing area and the fleet size are substantially smaller in Amsterdam/St. Paul (*ca* 3,220 km^2^ and 1 vessel respectively) than in SE Australia (*ca* 52,614 km^2^ and 5 vessels respectively), and therefore the density of vessels is higher in Amsterdam/St. Paul than in SE Australia (1 vessel for 10,523 km^2^). A smaller fishing area paired with a high density of vessels is likely to increase the probability of vessels being acoustically detected by whales if their natural distribution overlaps with that of fishing operations (Thode et al., 2007; Thode et al., 2015; Cruz et al., 2016), and to increase the predictability of the fishing activity (Guinet et al., 2014). A smaller fishing area may also limit the possibility for vessels implementing move-on strategies to avoid/escape depredation (Tixier et al., 2010; Tixier et al., 2014a). For example, an increased distance travelled by fishing vessels between fishing areas in response to killer whale interactions may significantly reduce their chances of being followed by the same whales (Tixier et al., 2010; present study). Therefore, with the two most distant fishing areas in SE Australia being 1,700 km apart and vessels able to travel distances >300 km between two successive fishing days (compared to 185 km between the two most distant fishing areas in Amsterdam/St. Paul), Australian vessels may have a broader range of options to outrun and/or avoid depredating killer whales. This assumption is further supported by the large spatial spread of interactions observed in the present study between Amsterdam/St. Paul (nearly 100% of the area) and SE Australia (<50%).

Fleet size, and more specifically the number of boats operating simultaneously in the same fishing area, has previously been shown to act as a dilution effect, where killer whales may opportunistically converge to any of the other vessels simultaneously operating nearby and, thereby, reduce the probability of individual vessels being depredated (Tixier et al., 2014a). As data from only one vessel were accessed in SE Australia out of fleet of 4–5 vessels, it is possible that such a dilution effect contributed to the observed lower killer whale interaction rate in SE Australia than Amsterdam/St. Paul. In contrast, in Amsterdam/St. Paul, killer whales have only one vessel to interact with and the detection, paired with the decisions made to reach or follow a vessel, may all converge to this single opportunity to depredate.

The correlation between the distance travelled by vessels and the probability of vessels to experience interactions with killer whales had a lower slope in Amsterdam/St. Paul than in SE Australia. While this difference may be explained by the dilution effect previously mentioned, with whales being more likely to follow the only fishing vessel, the total number of killer whales in the area potentially able to interact with vessels may also be a contributing factor. The probability of encountering new whales in subsequent fishing areas regardless of the distance travelled since the previous fishing may be highly dependent on the total number of depredating individuals in the region. While this study did not differentiate whether the same individuals followed vessels, the most recent assessment of the local killer whale population through photo-identification indicated a minimum of 63 depredating individuals around Amsterdam/St. Paul in 2016 (P Tixier, pers. comm., 2018). Paired with the small size of the Amsterdam/St. Paul area, this estimate indicates a high density of killer whales in this area, likely to increase the probability of the vessel to be detected by whales. The number of depredating killer whales is unknown in SE Australia. While preliminary studies suggested that a limited number of killer whale groups were involved in interactions with fishing vessels based on fishers’ observations (AFMA, 2005; Pease, 2012), the implementation of a consistent photo-identification effort from fishing vessels is needed to properly assess the total number of depredating individuals.

In addition to highlighting the level of depredation interactions, the results of the present study also provide preliminary insights into the ecology and movements of two poorly known killer whale populations. Previous studies have shown that most odontocete species/populations interacting with fishing vessels through depredation are naturally fish-eaters (Read, 2008; Hamer, Childerhouse & Gales, 2012). Hence, the fact that individuals from both populations in the present study depredate on blue-eye trevalla caught on longlines suggests that fish is a natural prey item in their diet. Indeed, killer whales have feeding ecologies and specialisation levels that vary greatly between populations. Individuals feeding exclusively on marine mammals have never been observed interacting with fishing vessels despite their distribution greatly overlapping with that of fishing operations (Matkin & Saulitis, 1994; Fearnbach et al., 2014; Peterson et al., 2014). In contrast, generalist feeding killer whales such as those present in Crozet waters (Guinet, 1992) or fish specialist killer whales in Alaska (Matkin & Saulitis, 1994; Fearnbach et al., 2014) are the ones being observed interacting with fisheries.

Killer whales around Amsterdam/St. Paul are regularly observed from platforms other than the fishing boat (i.e., from the shore of the islands and from a supply ship) but predation events on locally abundant marine mammals have never been observed or detected (C Guinet, pers. comm., 2018). In this area, fish resources are abundant at shallow depths (Beurois, 1975) and may be an important prey item of the local killer whale population. Such assumption is supported by sporadic observations of killer whales feeding on schooling fish (Emmelichthyidae) at the surface around Amsterdam/St. Paul (G Duhamel, pers. comm., 2018). In addition, the absence of effect of the daily fishing effort on the occurrence of interactions paired with interactions being reported in nearly 100% of the fishing area suggest a great overlap/proximity of the killer whale natural habitat and blue eye trevalla fishing operations in Amsterdam/St. Paul. Also, the absence of seasonality or annual trends in the proportion of fishing days with depredation around Amsterdam/St. Paul suggests that individuals from this population may remain in the area all year round over multiple years.

Information on the natural foraging ecology of killer whales in SE Australia is limited. Across this area, killer whales have been opportunistically documented feeding on both marine mammals, fish and sharks (Clode, 2004; Morrice, 2004; Naessig & Lanyon, 2004; Mustoe, 2008). Approximately 1,200 km to the west of the western-most fishing area in the present study, routinely monitored killer whales have been reported feeding on marine mammals, and possibly on fish and cephalopods (Wellard et al., 2016). However, whether these killer whales are the same individuals as those interacting with the blue-eye trevalla longline fishery to the east remains unknown. Interestingly, a seasonality (maximum in autumn, minimum in spring) was detected in the proportion of fishing days killer whales interacted with the blue-trevalla fishery in SE Australia which could result from temporal variations in their distribution and changes in their overlap with fishing areas (e.g., Söffker et al., 2015). Alternatively, such seasonality could be due to whales deciding whether or not to interact with vessels according to variations in natural prey availability (Tixier et al., 2016). The former of these two assumptions is supported by the consistency of the seasonal variations detected in the present study are consistent with those reported in opportunistic sightings from non-fishing vessels and from the shore in the north-eastern part the study area (Mustoe, 2008).

Whether this seasonality is driven by seasonal changes in the availability of blue eye trevalla remains unclear. Similar temporal patterns have been observed in other depredating killer whale populations for which the depredated resource is part of the natural diet of depredating whales (Passadore, Domingo & Secchi, 2015; Peterson & Hanselman, 2017). However, information on the ecology and seasonal patterns of blue eye trevalla stocks is limited. In SE Australia, landings are the greatest in late spring and summer months (Haddon, 2015), and this is likely mainly due to an increase in the fishing effort during this period by the vessel used in this study. As such, increased killer whale interaction rates in autumn and winter may correlate with periods of lower fishing effort, and possibly lower numbers of vessels of fleet operating simultaneously, therefore limiting the dilution effect mentioned above.

While further studies are needed to determine the extent to which the spatial distribution of killer whales overlaps with that of fishing vessels in SE Australia, the positive relationship between the daily fishing effort and the occurrence of interactions suggests that it may take time for killer whales to converge on the fishing gear once they detect a vessel. Paired with a large proportion of the fishing area where killer whale interactions have never been reported for the studied vessel over the 2010–2016 period (>50%), these findings suggest a limited extent of such overlap.

The variations of killer whale interaction levels within fisheries reported in the present study may also be driven by the spatial occurrence of fishing operations and the decisions made by captains (Richard et al., 2017). A captain effect was detected in SE Australia, with one of the two captains of the vessel used in the analyses experiencing higher interaction rates with killer whales. While this effect may potentially result from variations in the way data were collected between the two captains, it may also reflect different fishing behaviour and decisions. As such, lower frequencies of interactions during specific time of year and in recent years could be explained by captains targeting fishing areas of low probability of killer whale interactions and/or implementing effective strategies of avoidance when confronted by such interactions. For instance, the present study reported the distance travelled between consecutive fishing days and the fishing effort provided as two operational variables influencing the probability of killer whale interactions in SE Australia. Whether captains acting differently on these variables explains the observed differences may be used as a starting point to further assess the efficacy of specific fishing strategies in mitigating depredation.

The data used in this study were limited to only one vessel per fishery and, consequently, did not examine any vessel-related effects on killer whale interaction levels. Previous studies have emphasized large variations between vessels within fleets in the probability of interaction with odontocetes (Thode et al., 2007; Thode et al., 2015; Tixier et al., 2014a; Tixier et al., 2014b). For instance, while these variations may be due to the way captains use the gear, they may also depend on intrinsic factors such as the acoustic signature of vessels (Thode et al., 2015). Further analyses with finer scale data are, therefore, needed to elucidate the respective roles of vessels along with the fishing strategies of captains and the natural presence/movements of killer whales in the observed variations of whale-fishery interaction levels.

## Conclusion

In summary, the present study provided the first estimates of the frequency of killer whale depredation interactions in two commercial blue-eye trevalla longline fisheries in the Southern Hemisphere. Interactions were measured as a frequency of occurrence and further effort is now needed to assess the actual impact of these depredation interactions on the fishing catch, which is likely to have consequences for the fishing industry, the fish stocks and the local killer whale populations. The spatio-temporal patterns of these interactions were also examined and provided directions for future studies in identifying the respective roles of the behaviour of killer whales and fishing vessels in explaining these patterns. Finally, this study may help authorities to incorporate information on interactions with killer whales in the management of the fishing activity and the conservation of killer whale populations. For instance, measures to limit the amount of fish depredated by whales, such as changes in the fishing technique and temporary closures of the fishing activity, were recently implemented as regulations for the fishery operating in Amsterdam/St. Paul. In this area, which has been a national reserve since 2006, existing measures will be paired with the development and testing of protection devices for fish caught on hooks to limit killer whale depredation interactions.

##  Supplemental Information

10.7717/peerj.5306/supp-1 Data S1 Analysis of the occurrence of killer whale interactions Code for Generalised Linear Models fitted to the presence/absence of killer whales during fishing days

10.7717/peerj.5306/supp-2 Supplemental Information 1 Daily fishing operations and occurrence of killer whale interaction data used for Generalised Linear Models in Amsterdam/St. Paul and SE Australia

10.7717/peerj.5306/supp-3 Supplemental Information 2 Code (R Markdown) for the analysis used to model (Generalised Linear Models) the occurrence of killer whale interactions with longline fishing vessels in Amsterdam/St. Paul and SE Australia during fishing days

10.7717/peerj.5306/supp-4 Table S1 Predictors considered in the Generalised Linear Models fitted to the occurrence of killer whale interactions during fishing days, whether they were included as continuous terms or factors, their unit, range of values or levels

10.7717/peerj.5306/supp-5 Table S2 Summary output and parameter estimates of the best GLM-type model fitted with a binomial distribution on the presence/absence records of killer whale interaction with blue eye trevalla longline sets during fishing days Continuous predictors were the year, the latitude, the longitude, the depth at which vessels operated during fishing days, as well as the distance travelled from one day to the next and the daily fishing effort. The time of year vessels operated was tested through the *Season* as a categorical predictor, with the effects of *autumn*, *winter* and *spring* tested against the *summer* effect. The identity of the person who collected the data, which was a fishery observer in Amsterdam/St. Paul and the captain in SE Australia, was included as a categorical predictor. Outputs are provided for predictors that were included in the final model after a forward stepwise AIC selection, “ns” indicates that the predictor was not selected in the final model, and (-) indicates that the predictor was not tested.

## Additional Information and Declarations

### Data Availability

The following information was supplied regarding data availability: Raw data is supplied as Supplemental Materials.
