# Supplementary material for: Killer whale (Orcinus orca) interactions with blue-eye trevalla (Hyperoglyphe antarctica) longline fisheries
Source: PeerJ. 2018 Aug 8;6:e5306. doi: 10.7717/peerj.5306 (PMC6087417; doi:10.7717/peerj.5306)

DATA S1 - Tixier et al - killer whale - blue-eye trevalla

Paul Tixier

16 May 2018

# Analysis of the occurrence of killer whale interactions

### Code for Generalised Linear Models fitted to the presence/absence of killer whales per fishing day

Manuscript # 25457 " Killer whale (Orcinus orca) interactions with blue-eye trevalla (Hyperoglyphe antarctica) longline fisheries."

Paul Tixier1, Mary-Anne Lea2, Mark A. Hindell2, Christophe Guinet3, Nicolas Gasco4, Guy Duhamel4, John P.Y. Arnould1

1 Centre for Integrative Ecology, School of Life and Environmental Sciences, Deakin University, Burwood, Victoria, Australia 2 Ecology and Biodiversity Centre, Institute for Marine and Antarctic Studies, University of Tasmania, Hobart, Tasmania, Australia 3 Centre d'Etudes Biologiques de Chizé (CEBC), UMR 7372 Université de la Rochelle-CNRS, Villiers-en-Bois, France 4 Département Adaptations du vivant, UMR BOREA, Museum National d'Histoire Naturelle, Paris, France

Package Loading

## Data input

setwd("C:/Users/LES_research211/Documents/003_ANALYSES/011_BLUE_EYE_KIW")
data=read.csv("DATA_AUS_FRA2.csv",h=T,sep=",")

# Data exploration

Examine the data

str(data)

## 'data.frame': 12872 obs. of 33 variables:
## $ area : Factor w/ 2 levels "AUS","FRA": 1 1 1 1 1 1 1 1 1 1 ...
## $ vessel : Factor w/ 2 levels "AUS","PTE": 2 2 2 2 2 2 2 2 2 2 ...
## $ captain : Factor w/ 3 levels "BOB","PHIL","UNK": 1 1 1 1 1 1 1 1 1 1 ...
## $ observer : Factor w/ 9 levels "BEAF","ERIC",..: 9 9 9 9 9 9 9 9 9 9 ...
## $ trip : Factor w/ 256 levels "0 28 Apr Bass",..: 238 238 238 238 238 238 238 238 238 238 ...
## $ operation : int 1 2 3 4 5 6 7 9 8 11 ...
## $ technique : Factor w/ 6 levels "autoline","carrelet",..: 1 1 1 1 1 1 1 1 1 1 ...
## $ date : Factor w/ 1511 levels "2008/03/03","2008/03/04",..: 1 1 1 1 1 1 1 2 2 3 ...
## $ year : int 2008 2008 2008 2008 2008 2008 2008 2008 2008 2008 ...
## $ month : int 3 3 3 3 3 3 3 3 3 3 ...
## $ day : int 3 3 3 3 3 3 3 4 4 5 ...
## $ season : Factor w/ 4 levels "1_Summer","2_Autumn",..: 1 1 1 1 1 1 1 1 1 1 ...
## $ season2 : Factor w/ 4 levels "1_Summer","2_Autumn",..: 2 2 2 2 2 2 2 2 2 2 ...
## $ time : Factor w/ 918 levels "","0:00","0:01",..: 739 771 824 437 479 532 580 611 556 83 ...
## $ Lat_deg : int 36 36 36 36 36 36 36 36 36 36 ...
## $ Lat_min : num 54.9 52.3 48.6 45.7 44 41.9 38.8 40.8 36.8 38.7 ...
## $ Long_deg : num 137 137 137 137 137 137 137 136 136 136 ...
## $ Long_min : num 28.2 27.2 21.7 12.5 9.1 2.4 0.6 47 48.1 52.4 ...
## $ lat : num -36.9 -36.9 -36.8 -36.8 -36.7 ...
## $ lon : num 137 137 137 137 137 ...
## $ depth_min : int 381 311 376 281 315 391 333 322 255 351 ...
## $ depth_max : num 503 552 537 585 583 617 509 454 407 582 ...
## $ depth : num 442 432 456 433 449 ...
## $ effort : num 3 4 3 4 3 5 2 4 3 4 ...
## $ unit_effor : Factor w/ 5 levels "casier","hameçons",..: 4 4 4 4 4 4 4 4 4 4 ...
## $ effort_hook: num 3450 4600 3450 4600 3450 5750 2300 4600 3450 4600 ...
## $ length : num 2.17 3.31 2.54 3.24 2.48 3.65 1.59 3.23 2.43 3.51 ...
## $ lost : int 0 0 0 0 600 0 0 0 0 0 ...
## $ kiw_pres : int 0 0 0 0 0 0 0 0 0 0 ...
## $ spw_pres : int 0 0 0 0 0 0 0 0 0 0 ...
## $ piw_pres : int 0 0 0 0 0 0 0 0 0 0 ...
## $ subarea : Factor w/ 4 levels "AMS","BASS/TAS",..: 3 3 3 3 3 3 3 3 3 3 ...
## $ subarea3 : Factor w/ 4 levels "","BASS","SA",..: 3 3 3 3 3 3 3 3 3 3 ...

Examine a table of records (sets) by fishery and year

with(data, table(year, area))

## area
## year AUS FRA
## 2008 371 0
## 2009 318 0
## 2010 594 93
## 2011 803 409
## 2012 711 461
## 2013 662 382
## 2014 1014 378
## 2015 846 2074
## 2016 828 1714
## 2017 92 1122

Select data for the period 2010-2016

d1 <- data[data$year %in% c(2010:2016),]

with(d1, table(year, kiw_pres, useNA="always"))

## kiw_pres
## year 0 1 <NA>
## 2010 490 197 0
## 2011 895 315 2
## 2012 1009 153 10
## 2013 922 117 5
## 2014 1184 208 0
## 2015 1698 706 516
## 2016 1625 895 22
## <NA> 0 0 0

d1$kiw_pres_z = ifelse(is.na(d1$kiw_pres),0,d1$kiw_pres)

Examine the gear type (technique).

with(d1, table(year, technique,kiw_pres_z))

## , , kiw_pres_z = 0
##
## technique
## year autoline carrelet casier ligne à main palangre verticale pots
## 2010 433 3 5 40 9 0
## 2011 595 29 61 94 118 0
## 2012 601 63 153 52 150 0
## 2013 603 12 205 51 56 0
## 2014 865 37 45 134 103 0
## 2015 738 58 0 561 857 0
## 2016 735 97 0 147 656 12
##
## , , kiw_pres_z = 1
##
## technique
## year autoline carrelet casier ligne à main palangre verticale pots
## 2010 161 0 0 0 36 0
## 2011 208 4 0 19 84 0
## 2012 110 0 0 5 38 0
## 2013 59 0 1 15 42 0
## 2014 149 0 0 0 59 0
## 2015 108 5 0 10 583 0
## 2016 93 1 0 1 800 0

d1 <- droplevels(d1[d1$technique %in% c("autoline", "palangre verticale"),])
d1 <-subset(d1,!is.na(effort_hook))
d1 <-subset(d1,effort_hook>=1)

with(d1, table(year, technique,kiw_pres_z))

## , , kiw_pres_z = 0
##
## technique
## year autoline palangre verticale
## 2010 433 9
## 2011 594 118
## 2012 599 144
## 2013 603 55
## 2014 864 101
## 2015 738 629
## 2016 733 656
##
## , , kiw_pres_z = 1
##
## technique
## year autoline palangre verticale
## 2010 161 36
## 2011 208 84
## 2012 110 38
## 2013 59 42
## 2014 149 59
## 2015 108 583
## 2016 92 800

number of longline sets per area

d2 <- d1 %>%
 group_by(area) %>%
 summarise(count=n())
d2

## # A tibble: 2 x 2
## area count
## <fctr> <int>
## 1 AUS 5451
## 2 FRA 3354

number of longline sets per year

d4 <- d1 %>%
 group_by(area,year) %>%
 summarise(count=n())

d4_sum <- summarySE(d4,measurevar="count",groupvars="area")
d4_sum

## area N count sd se ci
## 1 AUS 7 778.7143 138.2651 52.2593 127.8739
## 2 FRA 7 479.1429 590.5925 223.2230 546.2069

number of longline sets per day

d3 <- d1 %>%
 group_by(area,year,date) %>%
 summarise(count=n())

d3_sum <- summarySE(d3,measurevar="count",groupvars="area")
d3_sum

## area N count sd se ci
## 1 AUS 1095 4.978082 1.921559 0.05806931 0.1139398
## 2 FRA 241 13.917012 14.667757 0.94483326 1.8612248

number of hooks per set

d5_sum <- summarySE(d1,measurevar="effort_hook",groupvars="area")
d5_sum

## area N effort_hook sd se ci
## 1 AUS 5451 2918.3755 1449.4262 19.631704 38.48598
## 2 FRA 3354 135.9958 194.9969 3.367024 6.60163

## B. Analysis

Data input

setwd("C:/Users/LES_research211/Documents/003_ANALYSES/011_BLUE_EYE_KIW")
data=read.csv("DATA_AUS_FRA2.csv",h=T,sep=",")
data <- droplevels(data[data$technique %in% c("autoline" ,"palangre verticale"),])
data <- droplevels(data[data$year %in% c("2010" ,"2011", "2012", "2013", "2014", "2015", "2016"),])
d1 <- droplevels(data)

# Calculate response variable

Presence/absence of killer whales during fishing days

dd_kw <- d1 %>%
 group_by(area,date) %>%
 summarise(kiw_pres= ifelse(any(kiw_pres==1),1, ifelse(all(kiw_pres==0),0,NA)))
dd_kw$area_day = paste(dd_kw$area,dd_kw$date)

## Calculated covariates

Calculate area_size, the total number of 0.1x0.1 degree cells in which each vessel operated in a trip. The first step is to assign sets to 0.1x0.1 degree cells

Define a function to allocate cells based on location

ji <- function(xy, origin=c(0,0), cellsize=c(0.1,0.1)) {
 t(apply(xy, 1, function(z) cellsize/2+origin+cellsize*(floor((z - origin)/cellsize))))
}
JI <- ji(cbind(d1$lon, d1$lat))
d1$X <- JI[, 1]
d1$Y <- JI[, 2]
d1$Cell <- paste(d1$X,"//", d1$Y)

Calculate the area_size for each vessel per day

dd_are <- d1 %>%
 group_by(area,date) %>%
 summarise(area_size = length(unique(Cell)))
dd_are$area_day = paste(dd_are$area,dd_are$date)

Calculate mean depth of sets per day

dd_depth <- d1 %>%
 group_by(area,date) %>%
 summarise(depth= mean(depth,na.rm=TRUE))
dd_depth$area_day = paste(dd_depth$area,dd_depth$date)

Calculate mean latitude per day

dd_lat <- d1 %>%
 group_by(area,date) %>%
 summarise(lat= mean(lat,na.rm=TRUE))
dd_lat$area_day = paste(dd_lat$area,dd_lat$date)

Calculate mean longitude per day

dd_lon <- d1 %>%
 group_by(area,date) %>%
 summarise(long= mean(lon,na.rm=TRUE))
dd_lon$area_day = paste(dd_lon$area,dd_lon$date)

Calculate sum of effort during fishing days

dd_eff <- d1 %>%
 group_by(area,date) %>%
 summarise(effort= sum(effort_hook,na.rm=TRUE))
dd_eff$area_day = paste(dd_eff$area,dd_eff$date)

Build the data frame with variables needed for modelling

d1$area_day = paste(d1$area,d1$date)
dd_kw$depth <- dd_depth$depth[match(dd_kw$area_day,dd_depth$area_day)]
dd_kw$lat <- dd_lat$lat[match(dd_kw$area_day,dd_lat$area_day)]
dd_kw$long <- dd_lon$long[match(dd_kw$area_day,dd_lon$area_day)]
dd_kw$area_size <- dd_are$area_size[match(dd_kw$area_day,dd_are$area_day)]
dd_kw$effort <- dd_eff$effort[match(dd_kw$area_day,dd_eff$area_day)]
dd_kw$captain <- d1$captain[match(dd_kw$area_day,d1$area_day)]
dd_kw$trip <- d1$trip[match(dd_kw$area_day,d1$area_day)]
dd_kw$vessel <- d1$vessel[match(dd_kw$area_day,d1$area_day)]
dd_kw$year <- d1$year[match(dd_kw$area_day,d1$area_day)]
dd_kw$season <- d1$season2[match(dd_kw$area_day,d1$area_day)]
dd_kw$observer <- d1$observer[match(dd_kw$area_day,d1$area_day)]
str(dd_kw)

## Classes 'grouped_df', 'tbl_df', 'tbl' and 'data.frame': 1338 obs. of 15 variables:
## $ area : Factor w/ 2 levels "AUS","FRA": 1 1 1 1 1 1 1 1 1 1 ...
## $ date : Factor w/ 1200 levels "2010/01/07","2010/01/08",..: 1 2 3 4 5 6 7 8 9 10 ...
## $ kiw_pres : num 0 1 0 0 1 0 0 0 0 0 ...
## $ area_day : chr "AUS 2010/01/07" "AUS 2010/01/08" "AUS 2010/01/12" "AUS 2010/01/13" ...
## $ depth : num 452 418 442 382 439 ...
## $ lat : num -40.8 -40.2 -37.7 -37.3 -37.1 ...
## $ long : num 149 149 140 139 138 ...
## $ area_size: int 1 3 2 5 3 2 3 2 2 2 ...
## $ effort : num 6325 13800 15295 14950 13800 ...
## $ captain : Factor w/ 3 levels "BOB","PHIL","UNK": 1 1 1 1 1 1 1 1 1 1 ...
## $ trip : Factor w/ 223 levels "0 28 Apr Bass",..: 215 215 216 216 216 216 216 217 217 217 ...
## $ vessel : Factor w/ 2 levels "AUS","PTE": 2 2 2 2 2 2 2 2 2 2 ...
## $ year : int 2010 2010 2010 2010 2010 2010 2010 2010 2010 2010 ...
## $ season : Factor w/ 4 levels "1_Summer","2_Autumn",..: 1 1 1 1 1 1 1 1 1 1 ...
## $ observer : Factor w/ 7 levels "ERIC","MAKA",..: 7 7 7 7 7 7 7 7 7 7 ...
## - attr(*, "vars")= chr "area"
## - attr(*, "drop")= logi TRUE

Remove days with kiw_pres == NA

dd_kw <-subset(dd_kw,!is.na(kiw_pres))
str(dd_kw) #(n = 4 days removed)

## Classes 'grouped_df', 'tbl_df', 'tbl' and 'data.frame': 1334 obs. of 15 variables:
## $ area : Factor w/ 2 levels "AUS","FRA": 1 1 1 1 1 1 1 1 1 1 ...
## $ date : Factor w/ 1200 levels "2010/01/07","2010/01/08",..: 1 2 3 4 5 6 7 8 9 10 ...
## $ kiw_pres : num 0 1 0 0 1 0 0 0 0 0 ...
## $ area_day : chr "AUS 2010/01/07" "AUS 2010/01/08" "AUS 2010/01/12" "AUS 2010/01/13" ...
## $ depth : num 452 418 442 382 439 ...
## $ lat : num -40.8 -40.2 -37.7 -37.3 -37.1 ...
## $ long : num 149 149 140 139 138 ...
## $ area_size: int 1 3 2 5 3 2 3 2 2 2 ...
## $ effort : num 6325 13800 15295 14950 13800 ...
## $ captain : Factor w/ 3 levels "BOB","PHIL","UNK": 1 1 1 1 1 1 1 1 1 1 ...
## $ trip : Factor w/ 223 levels "0 28 Apr Bass",..: 215 215 216 216 216 216 216 217 217 217 ...
## $ vessel : Factor w/ 2 levels "AUS","PTE": 2 2 2 2 2 2 2 2 2 2 ...
## $ year : int 2010 2010 2010 2010 2010 2010 2010 2010 2010 2010 ...
## $ season : Factor w/ 4 levels "1_Summer","2_Autumn",..: 1 1 1 1 1 1 1 1 1 1 ...
## $ observer : Factor w/ 7 levels "ERIC","MAKA",..: 7 7 7 7 7 7 7 7 7 7 ...
## - attr(*, "vars")= chr "area"
## - attr(*, "drop")= logi TRUE
## - attr(*, "indices")=List of 2
## ..$ : int 0 1 2 3 4 5 6 7 8 9 ...
## ..$ : int 1092 1093 1094 1095 1096 1097 1098 1099 1100 1101 ...
## - attr(*, "group_sizes")= int 1092 242
## - attr(*, "biggest_group_size")= int 1092
## - attr(*, "labels")='data.frame': 2 obs. of 1 variable:
## ..$ area: Factor w/ 2 levels "AUS","FRA": 1 2
## ..- attr(*, "vars")= chr "area"
## ..- attr(*, "drop")= logi TRUE

Create lagged variable = killer whale presence on previous day

n <- nrow(dd_kw)-1
d2 <- transform(dd_kw,kiw_pres_lag1=c(NA,kiw_pres[1:n]))

Calculate distance in km between consecutive fishing days

# Prepare data for modelling

Convert year as a continuous term

d1$year=as.numeric(as.character(d1$year))
with(d1, table(year, area))

## area
## year AUS FRA
## 2010 138 2
## 2011 184 15
## 2012 161 2
## 2013 139 1
## 2014 184 0
## 2015 146 45
## 2016 140 47

Convert latitudes into positive vales

d1$lat <- -(d1$lat)

# GLMs using stepwise forward selection

# Amsterdam/St Paul

Select data from Amsterdam/St Paul

d1_fr <- subset(d1, d1$area=="FRA")

Range/histogram of mean latitudes per day

range(d1_fr$lat)

## [1] 37.66333 39.29092

hist(d1_fr$lat)


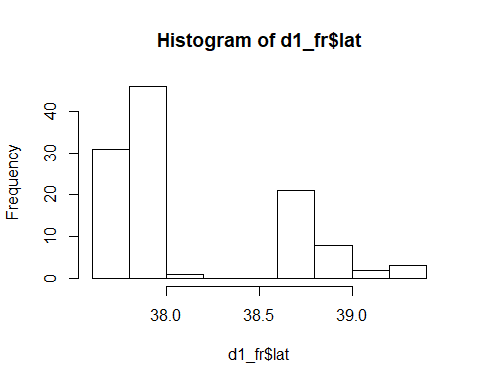


Range/histogram of mean longitudes per day

range(d1_fr$long)

## [1] 75.61035 77.89000

hist(d1_fr$long)


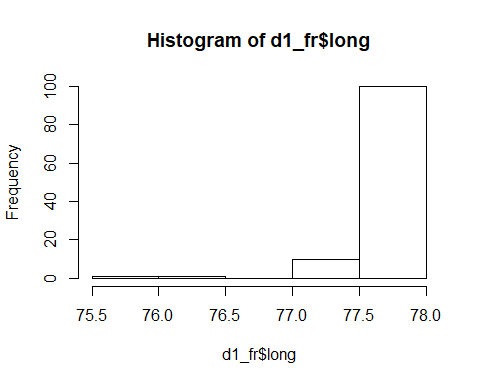


Range/histogram of mean depths per day

range(d1_fr$depth)

## [1] 55.47619 697.16667

hist(d1_fr$depth)


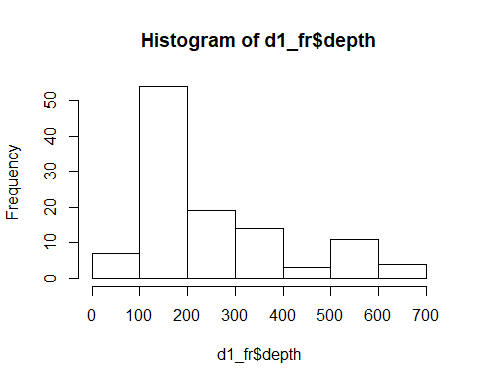


Range/histogram of effort per day

range(d1_fr$effort)

## [1] 400 7875

hist(d1_fr$effort)


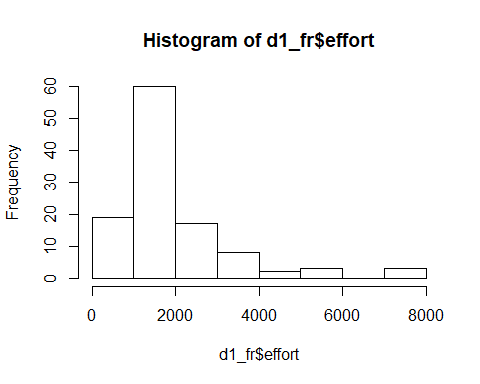


Range/histogram of size area fished per day

range(d1_fr$area_size)

## [1] 1 5

hist(d1_fr$area_size)


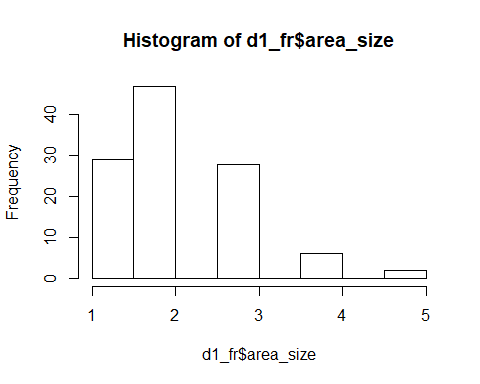


Range/histogram of distances from previous day

range(d1_fr$distance)

## [1] NA NA

hist(d1_fr$distance)


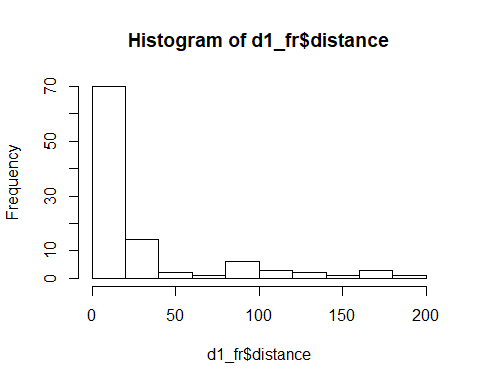


rescale all continuous terms

datsc_fr <- d1_fr
datsc_fr$distance <- as.numeric(scale(datsc_fr$distance))
datsc_fr$lat <- as.numeric(scale(datsc_fr$lat))
datsc_fr$year <- as.numeric(scale(datsc_fr$year))
datsc_fr$long <- as.numeric(scale(datsc_fr$long))
datsc_fr$depth <- as.numeric(scale(datsc_fr$depth))
datsc_fr$area_size <- as.numeric(scale(datsc_fr$area_size))
datsc_fr$effort <- as.numeric(scale(datsc_fr$effort))

Null model

glm_null_0 <- glm(kiw_pres ~ 1,
 family=binomial(link = "logit"), data=datsc_fr)
summary(glm_null_0)

##
## Call:
## glm(formula = kiw_pres ~ 1, family = binomial(link = "logit"),
## data = datsc_fr)
##
## Deviance Residuals:
## Min 1Q Median 3Q Max
## -1.4703 -1.4703 0.9104 0.9104 0.9104
##
## Coefficients:
## Estimate Std. Error z value Pr(>|z|)
## (Intercept) 0.6665 0.1996 3.34 0.000839 ***
## ---
## Signif. codes: 0 '***' 0.001 '**' 0.01 '*' 0.05 '.' 0.1 ' ' 1
##
## (Dispersion parameter for binomial family taken to be 1)
##
## Null deviance: 143.49 on 111 degrees of freedom
## Residual deviance: 143.49 on 111 degrees of freedom
## AIC: 145.49
##
## Number of Fisher Scoring iterations: 4

Check for temporal autocorrelation

acf(resid(glm_null_0, type="pearson"), plot=F, type="correlation")

##
## Autocorrelations of series 'resid(glm_null_0, type = "pearson")', by lag
##
## 0 1 2 3 4 5 6 7 8 9
## 1.000 0.438 0.154 0.044 -0.027 -0.058 -0.062 0.092 0.128 0.004
## 10 11 12 13 14 15 16 17 18 19
## -0.014 -0.005 0.044 -0.040 -0.005 0.044 -0.001 -0.032 -0.049 -0.040
## 20
## -0.111

acf(resid(glm_null_0, type="pearson"), plot=T, type="correlation")


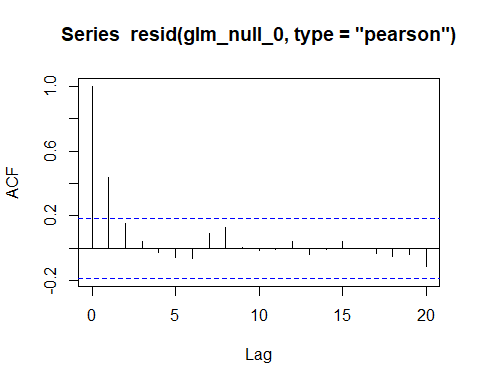


Null model with presence of killer whales during previous day in interaction with the distance travelled by vessels as a structural interaction term in the null model

glm_null <- glm(kiw_pres ~ kiw_pres_lag1*distance,
 family=binomial(link = "logit"), data=datsc_fr)
summary(glm_null)

##
## Call:
## glm(formula = kiw_pres ~ kiw_pres_lag1 * distance, family = binomial(link = "logit"),
## data = datsc_fr)
##
## Deviance Residuals:
## Min 1Q Median 3Q Max
## -1.8725 -0.8636 0.6241 0.6557 1.5769
##
## Coefficients:
## Estimate Std. Error z value Pr(>|z|)
## (Intercept) -0.3504 0.3716 -0.943 0.345682
## kiw_pres_lag1 1.7876 0.4882 3.661 0.000251 ***
## distance 0.8279 0.5208 1.590 0.111888
## kiw_pres_lag1:distance -1.0160 0.5816 -1.747 0.080644 .
## ---
## Signif. codes: 0 '***' 0.001 '**' 0.01 '*' 0.05 '.' 0.1 ' ' 1
##
## (Dispersion parameter for binomial family taken to be 1)
##
## Null deviance: 133.31 on 102 degrees of freedom
## Residual deviance: 110.98 on 99 degrees of freedom
## (9 observations deleted due to missingness)
## AIC: 118.98
##
## Number of Fisher Scoring iterations: 4

Check for temporal autocorrelation

acf(resid(glm_null, type="pearson"), plot=F, type="correlation")

##
## Autocorrelations of series 'resid(glm_null, type = "pearson")', by lag
##
## 0 1 2 3 4 5 6 7 8 9
## 1.000 -0.057 0.032 -0.021 0.054 -0.017 -0.172 0.068 0.070 0.036
## 10 11 12 13 14 15 16 17 18 19
## 0.030 -0.026 0.038 -0.081 0.053 0.136 0.001 -0.080 0.028 0.016
## 20
## -0.079

acf(resid(glm_null, type="pearson"), plot=T, type="correlation")


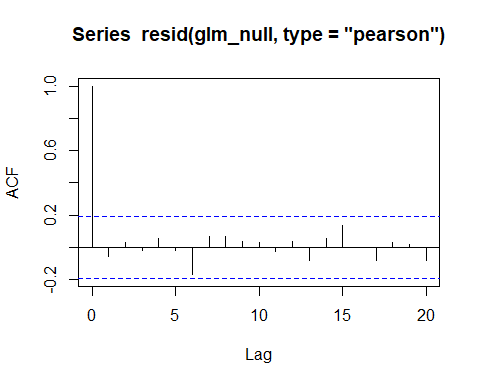


# Single term models

Add the year effect

glm_1.1 <- glm(kiw_pres ~ kiw_pres_lag1*distance + year,
 family=binomial(link = "logit"), data=datsc_fr)
summary(glm_1.1)

##
## Call:
## glm(formula = kiw_pres ~ kiw_pres_lag1 * distance + year, family = binomial(link = "logit"),
## data = datsc_fr)
##
## Deviance Residuals:
## Min 1Q Median 3Q Max
## -1.9470 -0.8835 0.5803 0.6482 1.8941
##
## Coefficients:
## Estimate Std. Error z value Pr(>|z|)
## (Intercept) -0.2804 0.3778 -0.742 0.457912
## kiw_pres_lag1 1.6681 0.4971 3.356 0.000791 ***
## distance 0.9719 0.5415 1.795 0.072667 .
## year 0.3257 0.2494 1.306 0.191573
## kiw_pres_lag1:distance -1.1440 0.6003 -1.906 0.056662 .
## ---
## Signif. codes: 0 '***' 0.001 '**' 0.01 '*' 0.05 '.' 0.1 ' ' 1
##
## (Dispersion parameter for binomial family taken to be 1)
##
## Null deviance: 133.31 on 102 degrees of freedom
## Residual deviance: 109.28 on 98 degrees of freedom
## (9 observations deleted due to missingness)
## AIC: 119.28
##
## Number of Fisher Scoring iterations: 4

Add the season effect

glm_1.2 <- glm(kiw_pres ~ kiw_pres_lag1*distance + season,
 family=binomial(link = "logit"), data=datsc_fr)
summary(glm_1.2)

##
## Call:
## glm(formula = kiw_pres ~ kiw_pres_lag1 * distance + season, family = binomial(link = "logit"),
## data = datsc_fr)
##
## Deviance Residuals:
## Min 1Q Median 3Q Max
## -1.8492 -0.8706 0.6352 0.6661 1.7404
##
## Coefficients:
## Estimate Std. Error z value Pr(>|z|)
## (Intercept) 14.7831 1385.3703 0.011 0.991486
## kiw_pres_lag1 1.7157 0.4905 3.498 0.000469 ***
## distance 0.9124 0.5470 1.668 0.095271 .
## season2_Autumn -15.0689 1385.3702 -0.011 0.991321
## season4_Spring -15.4401 1385.3704 -0.011 0.991108
## kiw_pres_lag1:distance -1.0370 0.5910 -1.755 0.079333 .
## ---
## Signif. codes: 0 '***' 0.001 '**' 0.01 '*' 0.05 '.' 0.1 ' ' 1
##
## (Dispersion parameter for binomial family taken to be 1)
##
## Null deviance: 133.31 on 102 degrees of freedom
## Residual deviance: 109.50 on 97 degrees of freedom
## (9 observations deleted due to missingness)
## AIC: 121.5
##
## Number of Fisher Scoring iterations: 15

Add the depth effect

glm_1.3 <- glm(kiw_pres ~ kiw_pres_lag1*distance + depth,
 family=binomial(link = "logit"), data=datsc_fr)
summary(glm_1.3)

##
## Call:
## glm(formula = kiw_pres ~ kiw_pres_lag1 * distance + depth, family = binomial(link = "logit"),
## data = datsc_fr)
##
## Deviance Residuals:
## Min 1Q Median 3Q Max
## -2.0300 -0.8336 0.6213 0.6815 1.6849
##
## Coefficients:
## Estimate Std. Error z value Pr(>|z|)
## (Intercept) -0.3484 0.3709 -0.939 0.347520
## kiw_pres_lag1 1.8243 0.4918 3.709 0.000208 ***
## distance 0.8766 0.5145 1.704 0.088457 .
## depth 0.2510 0.2565 0.978 0.327880
## kiw_pres_lag1:distance -1.1294 0.5858 -1.928 0.053887 .
## ---
## Signif. codes: 0 '***' 0.001 '**' 0.01 '*' 0.05 '.' 0.1 ' ' 1
##
## (Dispersion parameter for binomial family taken to be 1)
##
## Null deviance: 133.31 on 102 degrees of freedom
## Residual deviance: 109.98 on 98 degrees of freedom
## (9 observations deleted due to missingness)
## AIC: 119.98
##
## Number of Fisher Scoring iterations: 4

Add the latitude effect

glm_1.4 <- glm(kiw_pres ~ kiw_pres_lag1*distance + lat,
 family=binomial(link = "logit"), data=datsc_fr)
summary(glm_1.4)

##
## Call:
## glm(formula = kiw_pres ~ kiw_pres_lag1 * distance + lat, family = binomial(link = "logit"),
## data = datsc_fr)
##
## Deviance Residuals:
## Min 1Q Median 3Q Max
## -2.4093 -0.8337 0.4384 0.7361 1.6596
##
## Coefficients:
## Estimate Std. Error z value Pr(>|z|)
## (Intercept) -0.3001 0.3838 -0.782 0.434132
## kiw_pres_lag1 1.7261 0.5025 3.435 0.000592 ***
## distance 0.3181 0.5692 0.559 0.576256
## lat 0.6947 0.3035 2.289 0.022103 *
## kiw_pres_lag1:distance -0.6670 0.6141 -1.086 0.277420
## ---
## Signif. codes: 0 '***' 0.001 '**' 0.01 '*' 0.05 '.' 0.1 ' ' 1
##
## (Dispersion parameter for binomial family taken to be 1)
##
## Null deviance: 133.31 on 102 degrees of freedom
## Residual deviance: 104.85 on 98 degrees of freedom
## (9 observations deleted due to missingness)
## AIC: 114.85
##
## Number of Fisher Scoring iterations: 4

Add the longitude effect

glm_1.5 <- glm(kiw_pres ~ kiw_pres_lag1*distance + long,
 family=binomial(link = "logit"), data=datsc_fr)
summary(glm_1.5)

##
## Call:
## glm(formula = kiw_pres ~ kiw_pres_lag1 * distance + long, family = binomial(link = "logit"),
## data = datsc_fr)
##
## Deviance Residuals:
## Min 1Q Median 3Q Max
## -1.9026 -0.8874 0.6021 0.6672 1.6407
##
## Coefficients:
## Estimate Std. Error z value Pr(>|z|)
## (Intercept) -0.3041 0.3765 -0.808 0.41927
## kiw_pres_lag1 1.7157 0.4944 3.470 0.00052 ***
## distance 0.8040 0.5197 1.547 0.12181
## long -0.2714 0.3457 -0.785 0.43253
## kiw_pres_lag1:distance -1.0498 0.5840 -1.797 0.07226 .
## ---
## Signif. codes: 0 '***' 0.001 '**' 0.01 '*' 0.05 '.' 0.1 ' ' 1
##
## (Dispersion parameter for binomial family taken to be 1)
##
## Null deviance: 133.31 on 102 degrees of freedom
## Residual deviance: 110.13 on 98 degrees of freedom
## (9 observations deleted due to missingness)
## AIC: 120.13
##
## Number of Fisher Scoring iterations: 4

Add the total effort

glm_1.6 <- glm(kiw_pres ~ kiw_pres_lag1*distance + effort,
 family=binomial(link = "logit"), data=datsc_fr)
summary(glm_1.6)

##
## Call:
## glm(formula = kiw_pres ~ kiw_pres_lag1 * distance + effort, family = binomial(link = "logit"),
## data = datsc_fr)
##
## Deviance Residuals:
## Min 1Q Median 3Q Max
## -1.8794 -0.8790 0.6167 0.6663 1.7497
##
## Coefficients:
## Estimate Std. Error z value Pr(>|z|)
## (Intercept) -0.3337 0.3740 -0.892 0.372300
## kiw_pres_lag1 1.7627 0.4919 3.584 0.000339 ***
## distance 0.8845 0.5422 1.631 0.102838
## effort -0.0896 0.2376 -0.377 0.706164
## kiw_pres_lag1:distance -1.0662 0.5972 -1.785 0.074229 .
## ---
## Signif. codes: 0 '***' 0.001 '**' 0.01 '*' 0.05 '.' 0.1 ' ' 1
##
## (Dispersion parameter for binomial family taken to be 1)
##
## Null deviance: 133.31 on 102 degrees of freedom
## Residual deviance: 110.84 on 98 degrees of freedom
## (9 observations deleted due to missingness)
## AIC: 120.84
##
## Number of Fisher Scoring iterations: 4

Add the size of fished area

glm_1.7 <- glm(kiw_pres ~ kiw_pres_lag1*distance + area_size,
 family=binomial(link = "logit"), data=datsc_fr)
summary(glm_1.7)

##
## Call:
## glm(formula = kiw_pres ~ kiw_pres_lag1 * distance + area_size,
## family = binomial(link = "logit"), data = datsc_fr)
##
## Deviance Residuals:
## Min 1Q Median 3Q Max
## -2.0207 -0.8573 0.5614 0.7225 1.7277
##
## Coefficients:
## Estimate Std. Error z value Pr(>|z|)
## (Intercept) -0.3549 0.3762 -0.943 0.345480
## kiw_pres_lag1 1.8147 0.4952 3.665 0.000248 ***
## distance 0.7280 0.5328 1.366 0.171811
## area_size 0.3212 0.2467 1.302 0.192885
## kiw_pres_lag1:distance -0.9762 0.5922 -1.648 0.099269 .
## ---
## Signif. codes: 0 '***' 0.001 '**' 0.01 '*' 0.05 '.' 0.1 ' ' 1
##
## (Dispersion parameter for binomial family taken to be 1)
##
## Null deviance: 133.31 on 102 degrees of freedom
## Residual deviance: 109.22 on 98 degrees of freedom
## (9 observations deleted due to missingness)
## AIC: 119.22
##
## Number of Fisher Scoring iterations: 4

Model with the observer iD

glm_1.8 <- glm(kiw_pres ~ kiw_pres_lag1*distance + observer,
 family=binomial(link = "logit"), data=datsc_fr)
summary(glm_1.8)

##
## Call:
## glm(formula = kiw_pres ~ kiw_pres_lag1 * distance + observer,
## family = binomial(link = "logit"), data = datsc_fr)
##
## Deviance Residuals:
## Min 1Q Median 3Q Max
## -2.2639 -0.7366 0.4196 0.7595 1.9187
##
## Coefficients:
## Estimate Std. Error z value Pr(>|z|)
## (Intercept) 15.6178 1455.3980 0.011 0.99144
## kiw_pres_lag1 1.5421 0.5210 2.960 0.00308 **
## distance 1.1638 0.5908 1.970 0.04885 *
## observerMAKA -14.9767 1455.3978 -0.010 0.99179
## observerRBOC -15.5941 1455.3985 -0.011 0.99145
## observerSCAS -16.2866 1455.3980 -0.011 0.99107
## observerUNK -16.5086 1455.3981 -0.011 0.99095
## kiw_pres_lag1:distance -1.6278 0.6842 -2.379 0.01736 *
## ---
## Signif. codes: 0 '***' 0.001 '**' 0.01 '*' 0.05 '.' 0.1 ' ' 1
##
## (Dispersion parameter for binomial family taken to be 1)
##
## Null deviance: 133.31 on 102 degrees of freedom
## Residual deviance: 103.66 on 95 degrees of freedom
## (9 observations deleted due to missingness)
## AIC: 119.66
##
## Number of Fisher Scoring iterations: 14

### AIC summary

model.sel(list(glm_null, glm_1.1, glm_1.2, glm_1.3, glm_1.4, glm_1.5,glm_1.6,glm_1.7,glm_1.8))

## Model selection table
## (Int) dst kiw_prs_lg1 dst:kiw_prs_lg1 yer ssn dpt lat
## 5 -0.3001 0.3181 1.726 -0.6670 0.6947
## 1 -0.3504 0.8279 1.788 -1.0160
## 8 -0.3549 0.7280 1.815 -0.9762
## 2 -0.2804 0.9719 1.668 -1.1440 0.3257
## 4 -0.3484 0.8766 1.824 -1.1290 0.251
## 6 -0.3041 0.8040 1.716 -1.0500
## 9 15.6200 1.1640 1.542 -1.6280
## 7 -0.3337 0.8845 1.763 -1.0660
## 3 14.7800 0.9124 1.716 -1.0370 +
## lng eff are_siz obs df logLik AICc delta weight
## 5 5 -52.425 115.5 0.00 0.606
## 1 4 -55.490 119.4 3.92 0.085
## 8 0.3212 5 -54.608 119.8 4.37 0.068
## 2 5 -54.639 119.9 4.43 0.066
## 4 5 -54.991 120.6 5.13 0.047
## 6 -0.2714 5 -55.067 120.8 5.28 0.043
## 9 + 8 -51.828 121.2 5.72 0.035
## 7 -0.0896 5 -55.419 121.5 5.99 0.030
## 3 6 -54.750 122.4 6.91 0.019
## Models ranked by AICc(x)

Model with the latitude with lowest AIC

# Two terms models

glm_2.1 <- glm(kiw_pres ~ kiw_pres_lag1*distance + lat,
 family=binomial(link = "logit"), data=datsc_fr)
summary(glm_2.1)

##
## Call:
## glm(formula = kiw_pres ~ kiw_pres_lag1 * distance + lat, family = binomial(link = "logit"),
## data = datsc_fr)
##
## Deviance Residuals:
## Min 1Q Median 3Q Max
## -2.4093 -0.8337 0.4384 0.7361 1.6596
##
## Coefficients:
## Estimate Std. Error z value Pr(>|z|)
## (Intercept) -0.3001 0.3838 -0.782 0.434132
## kiw_pres_lag1 1.7261 0.5025 3.435 0.000592 ***
## distance 0.3181 0.5692 0.559 0.576256
## lat 0.6947 0.3035 2.289 0.022103 *
## kiw_pres_lag1:distance -0.6670 0.6141 -1.086 0.277420
## ---
## Signif. codes: 0 '***' 0.001 '**' 0.01 '*' 0.05 '.' 0.1 ' ' 1
##
## (Dispersion parameter for binomial family taken to be 1)
##
## Null deviance: 133.31 on 102 degrees of freedom
## Residual deviance: 104.85 on 98 degrees of freedom
## (9 observations deleted due to missingness)
## AIC: 114.85
##
## Number of Fisher Scoring iterations: 4

Model with the year

glm_2.2 <- glm(kiw_pres ~ kiw_pres_lag1*distance + lat + year,
 family=binomial(link = "logit"), data=datsc_fr)
summary(glm_2.2)

##
## Call:
## glm(formula = kiw_pres ~ kiw_pres_lag1 * distance + lat + year,
## family = binomial(link = "logit"), data = datsc_fr)
##
## Deviance Residuals:
## Min 1Q Median 3Q Max
## -2.4165 -0.8532 0.3802 0.6960 2.1570
##
## Coefficients:
## Estimate Std. Error z value Pr(>|z|)
## (Intercept) -0.1883 0.3925 -0.480 0.63148
## kiw_pres_lag1 1.5469 0.5162 2.997 0.00273 **
## distance 0.4571 0.5847 0.782 0.43432
## lat 0.8251 0.3271 2.523 0.01164 *
## year 0.5061 0.2824 1.792 0.07314 .
## kiw_pres_lag1:distance -0.8057 0.6273 -1.284 0.19897
## ---
## Signif. codes: 0 '***' 0.001 '**' 0.01 '*' 0.05 '.' 0.1 ' ' 1
##
## (Dispersion parameter for binomial family taken to be 1)
##
## Null deviance: 133.31 on 102 degrees of freedom
## Residual deviance: 101.49 on 97 degrees of freedom
## (9 observations deleted due to missingness)
## AIC: 113.49
##
## Number of Fisher Scoring iterations: 5

Model with the season

glm_2.3 <- glm(kiw_pres ~ kiw_pres_lag1*distance + lat + season,
 family=binomial(link = "logit"), data=datsc_fr)
summary(glm_2.3)

##
## Call:
## glm(formula = kiw_pres ~ kiw_pres_lag1 * distance + lat + season,
## family = binomial(link = "logit"), data = datsc_fr)
##
## Deviance Residuals:
## Min 1Q Median 3Q Max
## -2.3208 -0.8419 0.4167 0.7621 1.6526
##
## Coefficients:
## Estimate Std. Error z value Pr(>|z|)
## (Intercept) 15.2239 1385.2675 0.011 0.99123
## kiw_pres_lag1 1.6274 0.5055 3.220 0.00128 **
## distance 0.4163 0.5853 0.711 0.47692
## lat 0.7420 0.3081 2.408 0.01604 *
## season2_Autumn -15.4150 1385.2675 -0.011 0.99112
## season4_Spring -16.0078 1385.2677 -0.012 0.99078
## kiw_pres_lag1:distance -0.6555 0.6240 -1.050 0.29350
## ---
## Signif. codes: 0 '***' 0.001 '**' 0.01 '*' 0.05 '.' 0.1 ' ' 1
##
## (Dispersion parameter for binomial family taken to be 1)
##
## Null deviance: 133.31 on 102 degrees of freedom
## Residual deviance: 102.62 on 96 degrees of freedom
## (9 observations deleted due to missingness)
## AIC: 116.62
##
## Number of Fisher Scoring iterations: 15

Model with the depth

glm_2.4 <- glm(kiw_pres ~ kiw_pres_lag1*distance + lat + depth,
 family=binomial(link = "logit"), data=datsc_fr)
summary(glm_2.4)

##
## Call:
## glm(formula = kiw_pres ~ kiw_pres_lag1 * distance + lat + depth,
## family = binomial(link = "logit"), data = datsc_fr)
##
## Deviance Residuals:
## Min 1Q Median 3Q Max
## -2.3514 -0.8103 0.4431 0.7427 1.6424
##
## Coefficients:
## Estimate Std. Error z value Pr(>|z|)
## (Intercept) -0.2879 0.3835 -0.751 0.452919
## kiw_pres_lag1 1.7402 0.5027 3.462 0.000537 ***
## distance 0.3656 0.5669 0.645 0.518947
## lat 0.6830 0.3093 2.209 0.027199 *
## depth 0.2055 0.2715 0.757 0.449078
## kiw_pres_lag1:distance -0.7470 0.6204 -1.204 0.228605
## ---
## Signif. codes: 0 '***' 0.001 '**' 0.01 '*' 0.05 '.' 0.1 ' ' 1
##
## (Dispersion parameter for binomial family taken to be 1)
##
## Null deviance: 133.31 on 102 degrees of freedom
## Residual deviance: 104.26 on 97 degrees of freedom
## (9 observations deleted due to missingness)
## AIC: 116.26
##
## Number of Fisher Scoring iterations: 4

Model with the longitude

glm_2.5 <- glm(kiw_pres ~ kiw_pres_lag1*distance + lat + long,
 family=binomial(link = "logit"), data=datsc_fr)
summary(glm_2.5)

##
## Call:
## glm(formula = kiw_pres ~ kiw_pres_lag1 * distance + lat + long,
## family = binomial(link = "logit"), data = datsc_fr)
##
## Deviance Residuals:
## Min 1Q Median 3Q Max
## -2.3946 -0.8456 0.4255 0.7189 1.7803
##
## Coefficients:
## Estimate Std. Error z value Pr(>|z|)
## (Intercept) -0.2469 0.3884 -0.636 0.52486
## kiw_pres_lag1 1.6391 0.5094 3.217 0.00129 **
## distance 0.2626 0.5688 0.462 0.64435
## lat 0.7348 0.3068 2.395 0.01662 *
## long -0.3321 0.3223 -1.030 0.30292
## kiw_pres_lag1:distance -0.7409 0.6110 -1.213 0.22525
## ---
## Signif. codes: 0 '***' 0.001 '**' 0.01 '*' 0.05 '.' 0.1 ' ' 1
##
## (Dispersion parameter for binomial family taken to be 1)
##
## Null deviance: 133.31 on 102 degrees of freedom
## Residual deviance: 103.39 on 97 degrees of freedom
## (9 observations deleted due to missingness)
## AIC: 115.39
##
## Number of Fisher Scoring iterations: 4

Model with the total effort

glm_2.6 <- glm(kiw_pres ~ kiw_pres_lag1*distance + lat + effort,
 family=binomial(link = "logit"), data=datsc_fr)
summary(glm_2.6)

##
## Call:
## glm(formula = kiw_pres ~ kiw_pres_lag1 * distance + lat + effort,
## family = binomial(link = "logit"), data = datsc_fr)
##
## Deviance Residuals:
## Min 1Q Median 3Q Max
## -2.1916 -0.8608 0.3821 0.7114 1.8155
##
## Coefficients:
## Estimate Std. Error z value Pr(>|z|)
## (Intercept) -0.2066 0.3900 -0.530 0.5963
## kiw_pres_lag1 1.5996 0.5096 3.139 0.0017 **
## distance 0.4375 0.5711 0.766 0.4436
## lat 0.8967 0.3528 2.542 0.0110 *
## effort -0.4111 0.2813 -1.462 0.1438
## kiw_pres_lag1:distance -0.7931 0.6238 -1.271 0.2036
## ---
## Signif. codes: 0 '***' 0.001 '**' 0.01 '*' 0.05 '.' 0.1 ' ' 1
##
## (Dispersion parameter for binomial family taken to be 1)
##
## Null deviance: 133.31 on 102 degrees of freedom
## Residual deviance: 102.70 on 97 degrees of freedom
## (9 observations deleted due to missingness)
## AIC: 114.7
##
## Number of Fisher Scoring iterations: 5

Model with the size of fished area

glm_2.7 <- glm(kiw_pres ~ kiw_pres_lag1*distance + lat + area_size,
 family=binomial(link = "logit"), data=datsc_fr)
summary(glm_2.7)

##
## Call:
## glm(formula = kiw_pres ~ kiw_pres_lag1 * distance + lat + area_size,
## family = binomial(link = "logit"), data = datsc_fr)
##
## Deviance Residuals:
## Min 1Q Median 3Q Max
## -2.3363 -0.8248 0.4781 0.7154 1.7884
##
## Coefficients:
## Estimate Std. Error z value Pr(>|z|)
## (Intercept) -0.2950 0.3930 -0.751 0.452815
## kiw_pres_lag1 1.7409 0.5125 3.397 0.000682 ***
## distance 0.1518 0.5915 0.257 0.797446
## lat 0.7466 0.3127 2.388 0.016959 *
## area_size 0.4038 0.2671 1.512 0.130653
## kiw_pres_lag1:distance -0.6136 0.6193 -0.991 0.321716
## ---
## Signif. codes: 0 '***' 0.001 '**' 0.01 '*' 0.05 '.' 0.1 ' ' 1
##
## (Dispersion parameter for binomial family taken to be 1)
##
## Null deviance: 133.31 on 102 degrees of freedom
## Residual deviance: 102.42 on 97 degrees of freedom
## (9 observations deleted due to missingness)
## AIC: 114.42
##
## Number of Fisher Scoring iterations: 4

Model with the observer

glm_2.8 <- glm(kiw_pres ~ kiw_pres_lag1*distance + lat + observer,
 family=binomial(link = "logit"), data=datsc_fr)
summary(glm_2.8)

##
## Call:
## glm(formula = kiw_pres ~ kiw_pres_lag1 * distance + lat + observer,
## family = binomial(link = "logit"), data = datsc_fr)
##
## Deviance Residuals:
## Min 1Q Median 3Q Max
## -2.3195 -0.7016 0.4739 0.6775 1.8745
##
## Coefficients:
## Estimate Std. Error z value Pr(>|z|)
## (Intercept) 14.2375 1455.3982 0.010 0.99219
## kiw_pres_lag1 1.5062 0.5400 2.789 0.00528 **
## distance 0.6594 0.6325 1.043 0.29711
## lat 0.7149 0.3248 2.201 0.02775 *
## observerMAKA -13.5993 1455.3981 -0.009 0.99254
## observerRBOC -13.8681 1455.3988 -0.010 0.99240
## observerSCAS -14.8017 1455.3982 -0.010 0.99189
## observerUNK -15.3385 1455.3982 -0.011 0.99159
## kiw_pres_lag1:distance -1.2005 0.7116 -1.687 0.09159 .
## ---
## Signif. codes: 0 '***' 0.001 '**' 0.01 '*' 0.05 '.' 0.1 ' ' 1
##
## (Dispersion parameter for binomial family taken to be 1)
##
## Null deviance: 133.312 on 102 degrees of freedom
## Residual deviance: 97.954 on 94 degrees of freedom
## (9 observations deleted due to missingness)
## AIC: 115.95
##
## Number of Fisher Scoring iterations: 14

### AIC summary

model.sel(list(glm_2.1, glm_2.2, glm_2.3, glm_2.4, glm_2.5,glm_2.6,glm_2.7,glm_2.8))

## Model selection table
## (Int) dst kiw_prs_lg1 lat dst:kiw_prs_lg1 yer ssn dpt
## 2 -0.1883 0.4571 1.547 0.8251 -0.8057 0.5061
## 7 -0.2950 0.1518 1.741 0.7466 -0.6136
## 1 -0.3001 0.3181 1.726 0.6947 -0.6670
## 6 -0.2066 0.4375 1.600 0.8967 -0.7931
## 5 -0.2469 0.2626 1.639 0.7348 -0.7409
## 4 -0.2879 0.3656 1.740 0.6830 -0.7470 0.2055
## 3 15.2200 0.4163 1.627 0.7420 -0.6555 +
## 8 14.2400 0.6594 1.506 0.7149 -1.2000
## lng eff are_siz obs df logLik AICc delta weight
## 2 6 -50.746 114.4 0.00 0.267
## 7 0.4038 6 -51.212 115.3 0.93 0.168
## 1 5 -52.425 115.5 1.10 0.154
## 6 -0.4111 6 -51.349 115.6 1.21 0.146
## 5 -0.3321 6 -51.697 116.3 1.90 0.103
## 4 6 -52.128 117.1 2.76 0.067
## 3 7 -51.312 117.8 3.44 0.048
## 8 + 9 -48.977 117.9 3.52 0.046
## Models ranked by AICc(x)

Model with the year with lowest AIC

# Three terms models

glm_3.1 <- glm(kiw_pres ~ kiw_pres_lag1*distance + lat+year,
 family=binomial(link = "logit"), data=datsc_fr)
summary(glm_3.1)

##
## Call:
## glm(formula = kiw_pres ~ kiw_pres_lag1 * distance + lat + year,
## family = binomial(link = "logit"), data = datsc_fr)
##
## Deviance Residuals:
## Min 1Q Median 3Q Max
## -2.4165 -0.8532 0.3802 0.6960 2.1570
##
## Coefficients:
## Estimate Std. Error z value Pr(>|z|)
## (Intercept) -0.1883 0.3925 -0.480 0.63148
## kiw_pres_lag1 1.5469 0.5162 2.997 0.00273 **
## distance 0.4571 0.5847 0.782 0.43432
## lat 0.8251 0.3271 2.523 0.01164 *
## year 0.5061 0.2824 1.792 0.07314 .
## kiw_pres_lag1:distance -0.8057 0.6273 -1.284 0.19897
## ---
## Signif. codes: 0 '***' 0.001 '**' 0.01 '*' 0.05 '.' 0.1 ' ' 1
##
## (Dispersion parameter for binomial family taken to be 1)
##
## Null deviance: 133.31 on 102 degrees of freedom
## Residual deviance: 101.49 on 97 degrees of freedom
## (9 observations deleted due to missingness)
## AIC: 113.49
##
## Number of Fisher Scoring iterations: 5

Model with the season

glm_3.2 <- glm(kiw_pres ~ kiw_pres_lag1*distance + lat+year+season,
 family=binomial(link = "logit"), data=datsc_fr)
summary(glm_3.2)

##
## Call:
## glm(formula = kiw_pres ~ kiw_pres_lag1 * distance + lat + year +
## season, family = binomial(link = "logit"), data = datsc_fr)
##
## Deviance Residuals:
## Min 1Q Median 3Q Max
## -2.4075 -0.8346 0.3866 0.7114 2.3354
##
## Coefficients:
## Estimate Std. Error z value Pr(>|z|)
## (Intercept) 15.3203 1385.2407 0.011 0.99118
## kiw_pres_lag1 1.4277 0.5215 2.738 0.00619 **
## distance 0.3618 0.6007 0.602 0.54699
## lat 0.8667 0.3314 2.615 0.00892 **
## year 0.6775 0.3887 1.743 0.08133 .
## season2_Autumn -15.5973 1385.2407 -0.011 0.99102
## season4_Spring -14.9531 1385.2410 -0.011 0.99139
## kiw_pres_lag1:distance -0.7259 0.6300 -1.152 0.24923
## ---
## Signif. codes: 0 '***' 0.001 '**' 0.01 '*' 0.05 '.' 0.1 ' ' 1
##
## (Dispersion parameter for binomial family taken to be 1)
##
## Null deviance: 133.312 on 102 degrees of freedom
## Residual deviance: 99.367 on 95 degrees of freedom
## (9 observations deleted due to missingness)
## AIC: 115.37
##
## Number of Fisher Scoring iterations: 15

Model with the depth

glm_3.3 <- glm(kiw_pres ~ kiw_pres_lag1*distance + lat+year+depth,
 family=binomial(link = "logit"), data=datsc_fr)
summary(glm_3.3)

##
## Call:
## glm(formula = kiw_pres ~ kiw_pres_lag1 * distance + lat + year +
## depth, family = binomial(link = "logit"), data = datsc_fr)
##
## Deviance Residuals:
## Min 1Q Median 3Q Max
## -2.5956 -0.8657 0.3978 0.6982 2.1405
##
## Coefficients:
## Estimate Std. Error z value Pr(>|z|)
## (Intercept) -0.1669 0.3926 -0.425 0.6708
## kiw_pres_lag1 1.5588 0.5156 3.023 0.0025 **
## distance 0.4895 0.5782 0.847 0.3972
## lat 0.8314 0.3380 2.460 0.0139 *
## year 0.5190 0.2866 1.810 0.0702 .
## depth 0.2293 0.2813 0.815 0.4150
## kiw_pres_lag1:distance -0.8803 0.6292 -1.399 0.1618
## ---
## Signif. codes: 0 '***' 0.001 '**' 0.01 '*' 0.05 '.' 0.1 ' ' 1
##
## (Dispersion parameter for binomial family taken to be 1)
##
## Null deviance: 133.31 on 102 degrees of freedom
## Residual deviance: 100.80 on 96 degrees of freedom
## (9 observations deleted due to missingness)
## AIC: 114.8
##
## Number of Fisher Scoring iterations: 5

Model with the longitude

glm_3.4 <- glm(kiw_pres ~ kiw_pres_lag1*distance + lat+year+long,
 family=binomial(link = "logit"), data=datsc_fr)
summary(glm_3.4)

##
## Call:
## glm(formula = kiw_pres ~ kiw_pres_lag1 * distance + lat + year +
## long, family = binomial(link = "logit"), data = datsc_fr)
##
## Deviance Residuals:
## Min 1Q Median 3Q Max
## -2.3088 -0.8594 0.3654 0.7012 2.1781
##
## Coefficients:
## Estimate Std. Error z value Pr(>|z|)
## (Intercept) -0.1732 0.3933 -0.440 0.65965
## kiw_pres_lag1 1.5144 0.5189 2.918 0.00352 **
## distance 0.4129 0.5852 0.706 0.48045
## lat 0.8356 0.3261 2.562 0.01039 *
## year 0.4514 0.2923 1.545 0.12243
## long -0.2042 0.2948 -0.693 0.48853
## kiw_pres_lag1:distance -0.8500 0.6252 -1.360 0.17397
## ---
## Signif. codes: 0 '***' 0.001 '**' 0.01 '*' 0.05 '.' 0.1 ' ' 1
##
## (Dispersion parameter for binomial family taken to be 1)
##
## Null deviance: 133.31 on 102 degrees of freedom
## Residual deviance: 100.94 on 96 degrees of freedom
## (9 observations deleted due to missingness)
## AIC: 114.94
##
## Number of Fisher Scoring iterations: 5

Model with the effort

glm_3.5 <- glm(kiw_pres ~ kiw_pres_lag1*distance + lat+year+effort,
 family=binomial(link = "logit"), data=datsc_fr)
summary(glm_3.5)

##
## Call:
## glm(formula = kiw_pres ~ kiw_pres_lag1 * distance + lat + year +
## effort, family = binomial(link = "logit"), data = datsc_fr)
##
## Deviance Residuals:
## Min 1Q Median 3Q Max
## -2.3810 -0.8665 0.3673 0.7049 2.1282
##
## Coefficients:
## Estimate Std. Error z value Pr(>|z|)
## (Intercept) -0.1781 0.3938 -0.452 0.65103
## kiw_pres_lag1 1.5355 0.5168 2.971 0.00297 **
## distance 0.4732 0.5849 0.809 0.41850
## lat 0.8647 0.3533 2.448 0.01437 *
## year 0.4295 0.3712 1.157 0.24724
## effort -0.1203 0.3747 -0.321 0.74810
## kiw_pres_lag1:distance -0.8284 0.6314 -1.312 0.18950
## ---
## Signif. codes: 0 '***' 0.001 '**' 0.01 '*' 0.05 '.' 0.1 ' ' 1
##
## (Dispersion parameter for binomial family taken to be 1)
##
## Null deviance: 133.31 on 102 degrees of freedom
## Residual deviance: 101.39 on 96 degrees of freedom
## (9 observations deleted due to missingness)
## AIC: 115.39
##
## Number of Fisher Scoring iterations: 5

Model with the size of area fished

glm_3.6 <- glm(kiw_pres ~ kiw_pres_lag1*distance + lat+year+area_size,
 family=binomial(link = "logit"), data=datsc_fr)
summary(glm_3.6)

##
## Call:
## glm(formula = kiw_pres ~ kiw_pres_lag1 * distance + lat + year +
## area_size, family = binomial(link = "logit"), data = datsc_fr)
##
## Deviance Residuals:
## Min 1Q Median 3Q Max
## -2.5322 -0.8389 0.3887 0.6649 2.1394
##
## Coefficients:
## Estimate Std. Error z value Pr(>|z|)
## (Intercept) -0.1615 0.4057 -0.398 0.69051
## kiw_pres_lag1 1.5481 0.5265 2.940 0.00328 **
## distance 0.2870 0.6145 0.467 0.64042
## lat 0.8929 0.3440 2.596 0.00944 **
## year 0.4814 0.2820 1.707 0.08778 .
## area_size 0.3908 0.2776 1.408 0.15913
## kiw_pres_lag1:distance -0.7385 0.6428 -1.149 0.25060
## ---
## Signif. codes: 0 '***' 0.001 '**' 0.01 '*' 0.05 '.' 0.1 ' ' 1
##
## (Dispersion parameter for binomial family taken to be 1)
##
## Null deviance: 133.312 on 102 degrees of freedom
## Residual deviance: 99.392 on 96 degrees of freedom
## (9 observations deleted due to missingness)
## AIC: 113.39
##
## Number of Fisher Scoring iterations: 5

Model with the observer iD

glm_3.7 <- glm(kiw_pres ~ kiw_pres_lag1*distance + lat+year+observer,
 family=binomial(link = "logit"), data=datsc_fr)
summary(glm_3.7)

##
## Call:
## glm(formula = kiw_pres ~ kiw_pres_lag1 * distance + lat + year +
## observer, family = binomial(link = "logit"), data = datsc_fr)
##
## Deviance Residuals:
## Min 1Q Median 3Q Max
## -2.4180 -0.7032 0.4436 0.5619 1.8965
##
## Coefficients:
## Estimate Std. Error z value Pr(>|z|)
## (Intercept) 17.2943 1455.4008 0.012 0.9905
## kiw_pres_lag1 1.3350 0.5578 2.393 0.0167 *
## distance 0.8816 0.6489 1.359 0.1742
## lat 0.8013 0.3437 2.331 0.0197 *
## year 1.1951 1.0487 1.140 0.2544
## observerMAKA -17.3487 1455.4021 -0.012 0.9905
## observerRBOC -14.2045 1455.3989 -0.010 0.9922
## observerSCAS -17.8876 1455.4009 -0.012 0.9902
## observerUNK -16.2264 1455.3984 -0.011 0.9911
## kiw_pres_lag1:distance -1.3971 0.7238 -1.930 0.0536 .
## ---
## Signif. codes: 0 '***' 0.001 '**' 0.01 '*' 0.05 '.' 0.1 ' ' 1
##
## (Dispersion parameter for binomial family taken to be 1)
##
## Null deviance: 133.312 on 102 degrees of freedom
## Residual deviance: 96.081 on 93 degrees of freedom
## (9 observations deleted due to missingness)
## AIC: 116.08
##
## Number of Fisher Scoring iterations: 14

### AIC summary

model.sel(list(glm_3.1, glm_3.2, glm_3.3, glm_3.4, glm_3.5,glm_3.6,glm_3.7))

## Model selection table
## (Int) dst kiw_prs_lg1 lat yer dst:kiw_prs_lg1 ssn dpt
## 1 -0.1883 0.4571 1.547 0.8251 0.5061 -0.8057
## 6 -0.1615 0.2870 1.548 0.8929 0.4814 -0.7385
## 3 -0.1669 0.4895 1.559 0.8314 0.5190 -0.8803 0.2293
## 4 -0.1732 0.4129 1.514 0.8356 0.4514 -0.8500
## 5 -0.1781 0.4732 1.535 0.8647 0.4295 -0.8284
## 2 15.3200 0.3618 1.428 0.8667 0.6775 -0.7259 +
## 7 17.2900 0.8816 1.335 0.8013 1.1950 -1.3970
## lng eff are_siz obs df logLik AICc delta weight
## 1 6 -50.746 114.4 0.00 0.285
## 6 0.3908 7 -49.696 114.6 0.20 0.257
## 3 7 -50.400 116.0 1.61 0.127
## 4 -0.2042 7 -50.469 116.1 1.75 0.119
## 5 -0.1203 7 -50.695 116.6 2.20 0.095
## 2 8 -49.684 116.9 2.53 0.080
## 7 + 10 -48.041 118.5 4.10 0.037
## Models ranked by AICc(x)

None of the 3 terms models further improved the AIC The final model should be

glm_3.1 <- glm(kiw_pres ~ kiw_pres_lag1*distance + lat+year,
 family=binomial(link = "logit"), data=datsc_fr)
summary(glm_3.1)

##
## Call:
## glm(formula = kiw_pres ~ kiw_pres_lag1 * distance + lat + year,
## family = binomial(link = "logit"), data = datsc_fr)
##
## Deviance Residuals:
## Min 1Q Median 3Q Max
## -2.4165 -0.8532 0.3802 0.6960 2.1570
##
## Coefficients:
## Estimate Std. Error z value Pr(>|z|)
## (Intercept) -0.1883 0.3925 -0.480 0.63148
## kiw_pres_lag1 1.5469 0.5162 2.997 0.00273 **
## distance 0.4571 0.5847 0.782 0.43432
## lat 0.8251 0.3271 2.523 0.01164 *
## year 0.5061 0.2824 1.792 0.07314 .
## kiw_pres_lag1:distance -0.8057 0.6273 -1.284 0.19897
## ---
## Signif. codes: 0 '***' 0.001 '**' 0.01 '*' 0.05 '.' 0.1 ' ' 1
##
## (Dispersion parameter for binomial family taken to be 1)
##
## Null deviance: 133.31 on 102 degrees of freedom
## Residual deviance: 101.49 on 97 degrees of freedom
## (9 observations deleted due to missingness)
## AIC: 113.49
##
## Number of Fisher Scoring iterations: 5

# Model validation

plots of residuals versus fitted values for check of linearity and homoscedasticity of variance

plot(fitted(glm_3.1), resid(glm_3.1), main="Residual Plot for linearity and homoscedasticity of variance",xlab="Fitted value",ylab="Residuals")


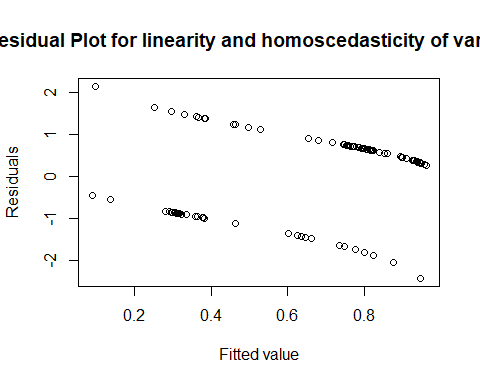


test for normality of the residuals

hist(resid(glm_3.1),xlab="Residuals",main="Normality of the Residuals")


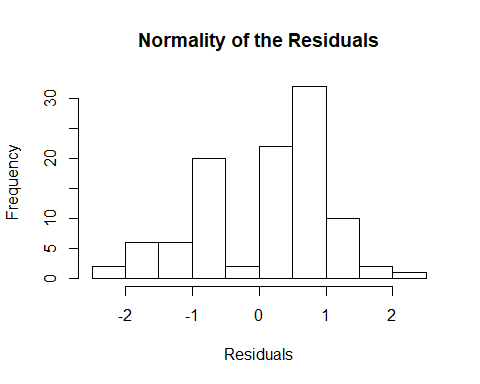


plot the residuals against each explanatory variables to test independency

d1_fr2 <- subset(d1_fr, !is.na(d1_fr$distance))
plot(resid(glm_3.1)~d1_fr2$distance,pch=20,main="Residual Plot for independency 1",xlab="Distance (km)",ylab="Residuals")


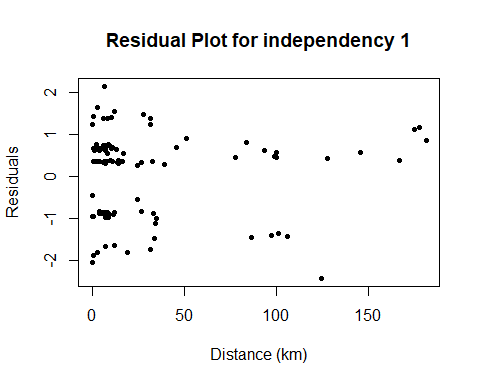


plot(resid(glm_3.1)~d1_fr2$lat,pch=20,main="Residual Plot for independency 2",xlab="Latitude",ylab="Residuals")


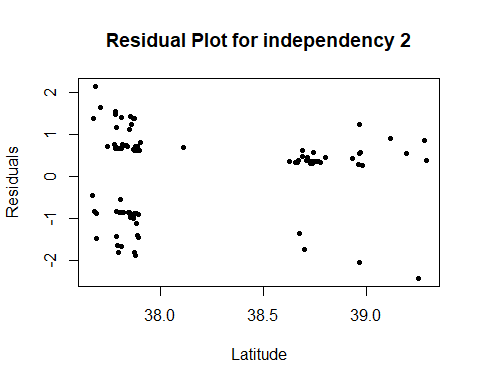


Check for temporal autocorrelation

acf(resid(glm_3.1, type="pearson"), plot=T, type="correlation")


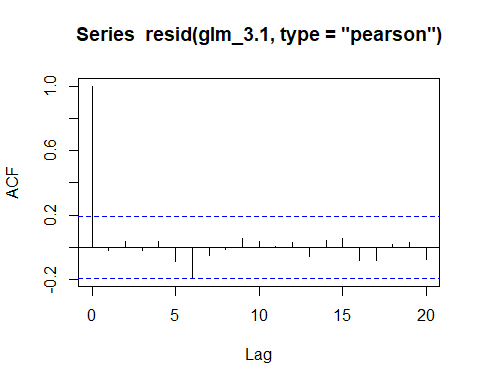


# SE Australia

Select data from SE Australia

d1_au <- subset(d1, d1$area=="AUS")

Range/histogram of mean latitudes per day

range(d1_au$lat)

## [1] 33.60667 44.15583

hist(d1_au$lat)


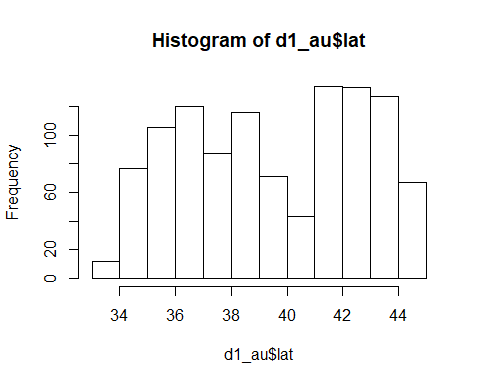


Range/histogram of mean longitudes per day

range(d1_au$long)

## [1] 131.6804 150.4473

hist(d1_au$long)


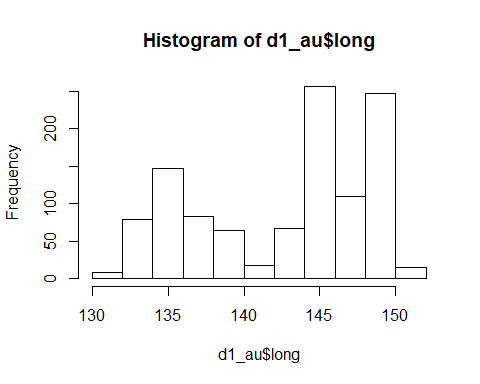


Range/histogram of mean depths per day

range(d1_au$depth, na.rm=TRUE)

## [1] 132.7143 892.9000

hist(d1_au$depth)


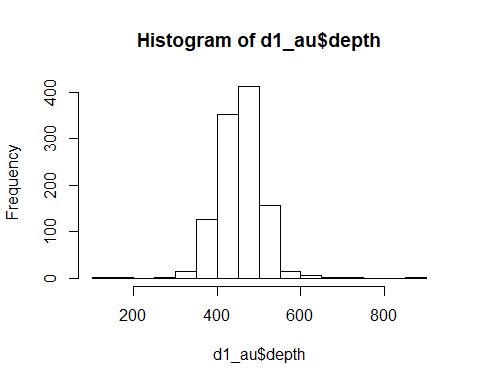


Range/histogram of effort per day

range(d1_au$effort)

## [1] 1150 29900

hist(d1_au$effort)


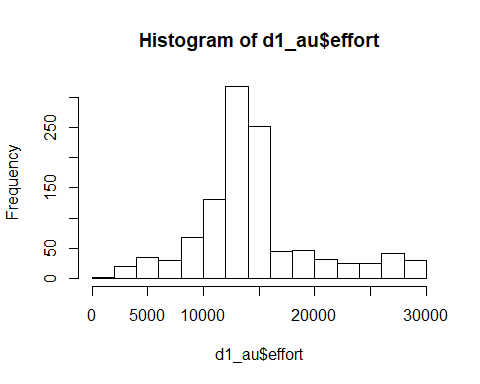


Range/histogram of size area fished per trip

range(d1_au$area_size)

## [1] 1 8

hist(d1_au$area_size)


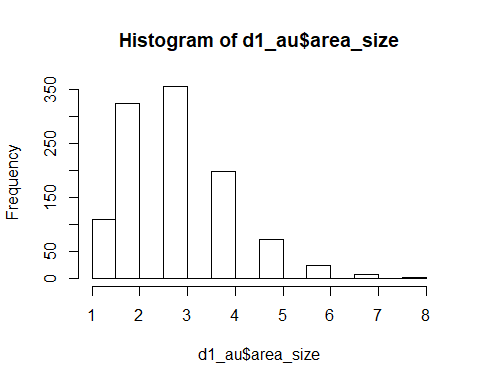


Range/histogram of distances from previous day

range(d1_au$distance,na.rm=TRUE)

## [1] 0.6917967 867.3561402

hist(d1_au$distance)


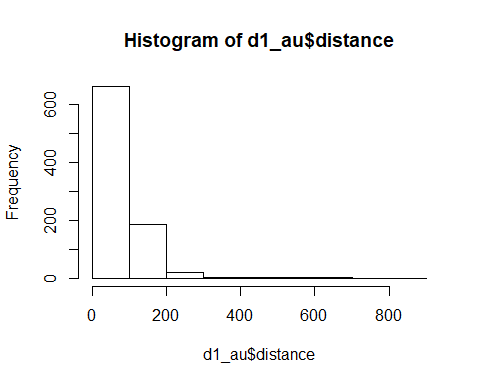


One extreme value of distance is removed from the data used for modeling

d1_au <- subset(d1_au, d1_au$distance<=800)
range(d1_au$distance,na.rm=TRUE)

## [1] 0.6917967 680.4216311

rescale all continuous terms

datsc_au <- d1_au
datsc_au$distance <- as.numeric(scale(datsc_au$distance))
datsc_au$lat <- as.numeric(scale(datsc_au$lat))
datsc_au$year <- as.numeric(scale(datsc_au$year))
datsc_au$long <- as.numeric(scale(datsc_au$long))
datsc_au$depth <- as.numeric(scale(datsc_au$depth))
datsc_au$area_size <- as.numeric(scale(datsc_au$area_size))
datsc_au$effort <- as.numeric(scale(datsc_au$effort))

Null model

glm_null_0 <- glm(kiw_pres ~ 1,
 family=binomial(link = "logit"), data=datsc_au)
summary(glm_null_0)

##
## Call:
## glm(formula = kiw_pres ~ 1, family = binomial(link = "logit"),
## data = datsc_au)
##
## Deviance Residuals:
## Min 1Q Median 3Q Max
## -0.7165 -0.7165 -0.7165 -0.7165 1.7236
##
## Coefficients:
## Estimate Std. Error z value Pr(>|z|)
## (Intercept) -1.2288 0.0806 -15.25 <2e-16 ***
## ---
## Signif. codes: 0 '***' 0.001 '**' 0.01 '*' 0.05 '.' 0.1 ' ' 1
##
## (Dispersion parameter for binomial family taken to be 1)
##
## Null deviance: 940.32 on 878 degrees of freedom
## Residual deviance: 940.32 on 878 degrees of freedom
## AIC: 942.32
##
## Number of Fisher Scoring iterations: 4

Check for temporal autocorrelation

acf(resid(glm_null_0, type="pearson"), plot=F, type="correlation")

##
## Autocorrelations of series 'resid(glm_null_0, type = "pearson")', by lag
##
## 0 1 2 3 4 5 6 7 8 9
## 1.000 0.449 0.299 0.190 0.092 0.053 0.040 0.052 0.071 0.013
## 10 11 12 13 14 15 16 17 18 19
## -0.007 -0.027 -0.034 -0.013 0.001 -0.024 -0.042 0.004 -0.003 -0.035
## 20 21 22 23 24 25 26 27 28 29
## -0.049 -0.043 0.017 0.017 -0.016 0.023 0.003 0.012 -0.034 -0.033

acf(resid(glm_null_0, type="pearson"), plot=T, type="correlation")


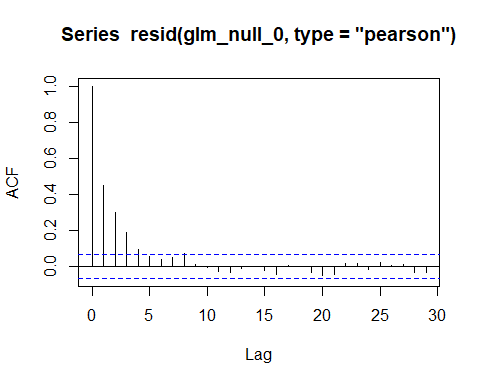


Presence of killer whales during previous day in interaction with the distance travelled by vessels as a structural interaction term in the null model

glm_null <- glm(kiw_pres ~ kiw_pres_lag1*distance,
 family=binomial(link = "logit"), data=datsc_au)
summary(glm_null)

##
## Call:
## glm(formula = kiw_pres ~ kiw_pres_lag1 * distance, family = binomial(link = "logit"),
## data = datsc_au)
##
## Deviance Residuals:
## Min 1Q Median 3Q Max
## -1.9613 -0.5411 -0.4951 -0.3584 2.3587
##
## Coefficients:
## Estimate Std. Error z value Pr(>|z|)
## (Intercept) -2.0433 0.1217 -16.791 < 2e-16 ***
## kiw_pres_lag1 2.6323 0.2042 12.889 < 2e-16 ***
## distance -0.3018 0.1570 -1.922 0.05460 .
## kiw_pres_lag1:distance -0.8376 0.3076 -2.723 0.00647 **
## ---
## Signif. codes: 0 '***' 0.001 '**' 0.01 '*' 0.05 '.' 0.1 ' ' 1
##
## (Dispersion parameter for binomial family taken to be 1)
##
## Null deviance: 940.32 on 878 degrees of freedom
## Residual deviance: 717.75 on 875 degrees of freedom
## AIC: 725.75
##
## Number of Fisher Scoring iterations: 5

Check for temporal autocorrelation

acf(resid(glm_null, type="pearson"), plot=F, type="correlation")

##
## Autocorrelations of series 'resid(glm_null, type = "pearson")', by lag
##
## 0 1 2 3 4 5 6 7 8 9
## 1.000 -0.027 0.106 0.064 0.016 -0.013 0.048 0.007 0.086 -0.023
## 10 11 12 13 14 15 16 17 18 19
## 0.001 -0.022 -0.026 0.013 0.012 -0.011 -0.073 -0.001 -0.004 -0.046
## 20 21 22 23 24 25 26 27 28 29
## -0.048 -0.052 0.003 -0.019 -0.059 0.010 -0.037 -0.012 0.025 -0.066

acf(resid(glm_null, type="pearson"), plot=T, type="correlation")


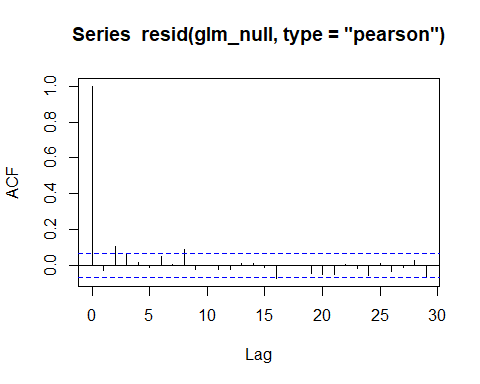


# Single term models

Add the year effect

glm_1.1 <- glm(kiw_pres ~ kiw_pres_lag1*distance + year,
 family=binomial(link = "logit"), data=datsc_au)
summary(glm_1.1)

##
## Call:
## glm(formula = kiw_pres ~ kiw_pres_lag1 * distance + year, family = binomial(link = "logit"),
## data = datsc_au)
##
## Deviance Residuals:
## Min 1Q Median 3Q Max
## -1.9584 -0.5409 -0.4952 -0.3620 2.3180
##
## Coefficients:
## Estimate Std. Error z value Pr(>|z|)
## (Intercept) -2.04066 0.12171 -16.767 < 2e-16 ***
## kiw_pres_lag1 2.61841 0.20540 12.748 < 2e-16 ***
## distance -0.28910 0.15799 -1.830 0.06727 .
## year -0.05659 0.09834 -0.576 0.56494
## kiw_pres_lag1:distance -0.82820 0.30714 -2.697 0.00701 **
## ---
## Signif. codes: 0 '***' 0.001 '**' 0.01 '*' 0.05 '.' 0.1 ' ' 1
##
## (Dispersion parameter for binomial family taken to be 1)
##
## Null deviance: 940.32 on 878 degrees of freedom
## Residual deviance: 717.42 on 874 degrees of freedom
## AIC: 727.42
##
## Number of Fisher Scoring iterations: 5

Add the season effect

glm_1.2 <- glm(kiw_pres ~ kiw_pres_lag1*distance + season,
 family=binomial(link = "logit"), data=datsc_au)
summary(glm_1.2)

##
## Call:
## glm(formula = kiw_pres ~ kiw_pres_lag1 * distance + season, family = binomial(link = "logit"),
## data = datsc_au)
##
## Deviance Residuals:
## Min 1Q Median 3Q Max
## -2.0300 -0.5509 -0.4777 -0.3490 2.3663
##
## Coefficients:
## Estimate Std. Error z value Pr(>|z|)
## (Intercept) -1.89718 0.17732 -10.699 < 2e-16 ***
## kiw_pres_lag1 2.61884 0.20534 12.754 < 2e-16 ***
## distance -0.30471 0.15762 -1.933 0.05321 .
## season2_Autumn 0.01615 0.24470 0.066 0.94739
## season3_Winter -0.25391 0.36813 -0.690 0.49037
## season4_Spring -0.40155 0.24124 -1.665 0.09600 .
## kiw_pres_lag1:distance -0.85984 0.30924 -2.781 0.00543 **
## ---
## Signif. codes: 0 '***' 0.001 '**' 0.01 '*' 0.05 '.' 0.1 ' ' 1
##
## (Dispersion parameter for binomial family taken to be 1)
##
## Null deviance: 940.32 on 878 degrees of freedom
## Residual deviance: 713.95 on 872 degrees of freedom
## AIC: 727.95
##
## Number of Fisher Scoring iterations: 5

Add the depth effect

glm_1.3 <- glm(kiw_pres ~ kiw_pres_lag1*distance + depth,
 family=binomial(link = "logit"), data=datsc_au)
summary(glm_1.3)

##
## Call:
## glm(formula = kiw_pres ~ kiw_pres_lag1 * distance + depth, family = binomial(link = "logit"),
## data = datsc_au)
##
## Deviance Residuals:
## Min 1Q Median 3Q Max
## -1.9647 -0.5397 -0.4949 -0.3644 2.3513
##
## Coefficients:
## Estimate Std. Error z value Pr(>|z|)
## (Intercept) -2.04470 0.12178 -16.790 < 2e-16 ***
## kiw_pres_lag1 2.63129 0.20454 12.865 < 2e-16 ***
## distance -0.30052 0.15688 -1.916 0.05542 .
## depth -0.05240 0.09697 -0.540 0.58893
## kiw_pres_lag1:distance -0.82655 0.30743 -2.689 0.00718 **
## ---
## Signif. codes: 0 '***' 0.001 '**' 0.01 '*' 0.05 '.' 0.1 ' ' 1
##
## (Dispersion parameter for binomial family taken to be 1)
##
## Null deviance: 937.35 on 877 degrees of freedom
## Residual deviance: 717.12 on 873 degrees of freedom
## (1 observation deleted due to missingness)
## AIC: 727.12
##
## Number of Fisher Scoring iterations: 5

Add the latitude effect

glm_1.4 <- glm(kiw_pres ~ kiw_pres_lag1*distance + lat,
 family=binomial(link = "logit"), data=datsc_au)
summary(glm_1.4)

##
## Call:
## glm(formula = kiw_pres ~ kiw_pres_lag1 * distance + lat, family = binomial(link = "logit"),
## data = datsc_au)
##
## Deviance Residuals:
## Min 1Q Median 3Q Max
## -1.9505 -0.5464 -0.4814 -0.3454 2.3884
##
## Coefficients:
## Estimate Std. Error z value Pr(>|z|)
## (Intercept) -2.04691 0.12216 -16.756 < 2e-16 ***
## kiw_pres_lag1 2.60661 0.20478 12.729 < 2e-16 ***
## distance -0.30250 0.15806 -1.914 0.05563 .
## lat 0.16735 0.09589 1.745 0.08095 .
## kiw_pres_lag1:distance -0.89287 0.31299 -2.853 0.00434 **
## ---
## Signif. codes: 0 '***' 0.001 '**' 0.01 '*' 0.05 '.' 0.1 ' ' 1
##
## (Dispersion parameter for binomial family taken to be 1)
##
## Null deviance: 940.32 on 878 degrees of freedom
## Residual deviance: 714.68 on 874 degrees of freedom
## AIC: 724.68
##
## Number of Fisher Scoring iterations: 5

Add the longitude effect

glm_1.5 <- glm(kiw_pres ~ kiw_pres_lag1*distance + long,
 family=binomial(link = "logit"), data=datsc_au)
summary(glm_1.5)

##
## Call:
## glm(formula = kiw_pres ~ kiw_pres_lag1 * distance + long, family = binomial(link = "logit"),
## data = datsc_au)
##
## Deviance Residuals:
## Min 1Q Median 3Q Max
## -1.9876 -0.5388 -0.4970 -0.3571 2.3455
##
## Coefficients:
## Estimate Std. Error z value Pr(>|z|)
## (Intercept) -2.04351 0.12174 -16.786 <2e-16 ***
## kiw_pres_lag1 2.62823 0.20432 12.863 <2e-16 ***
## distance -0.30264 0.15726 -1.924 0.0543 .
## long 0.05192 0.09646 0.538 0.5904
## kiw_pres_lag1:distance -0.84371 0.30824 -2.737 0.0062 **
## ---
## Signif. codes: 0 '***' 0.001 '**' 0.01 '*' 0.05 '.' 0.1 ' ' 1
##
## (Dispersion parameter for binomial family taken to be 1)
##
## Null deviance: 940.32 on 878 degrees of freedom
## Residual deviance: 717.46 on 874 degrees of freedom
## AIC: 727.46
##
## Number of Fisher Scoring iterations: 5

Add the total effort

glm_1.6 <- glm(kiw_pres ~ kiw_pres_lag1*distance + effort,
 family=binomial(link = "logit"), data=datsc_au)
summary(glm_1.6)

##
## Call:
## glm(formula = kiw_pres ~ kiw_pres_lag1 * distance + effort, family = binomial(link = "logit"),
## data = datsc_au)
##
## Deviance Residuals:
## Min 1Q Median 3Q Max
## -2.1549 -0.5334 -0.4665 -0.3228 2.4608
##
## Coefficients:
## Estimate Std. Error z value Pr(>|z|)
## (Intercept) -2.08899 0.12513 -16.695 < 2e-16 ***
## kiw_pres_lag1 2.70738 0.20971 12.910 < 2e-16 ***
## distance -0.34280 0.16192 -2.117 0.034248 *
## effort 0.32497 0.09274 3.504 0.000458 ***
## kiw_pres_lag1:distance -0.87633 0.31177 -2.811 0.004942 **
## ---
## Signif. codes: 0 '***' 0.001 '**' 0.01 '*' 0.05 '.' 0.1 ' ' 1
##
## (Dispersion parameter for binomial family taken to be 1)
##
## Null deviance: 940.32 on 878 degrees of freedom
## Residual deviance: 705.61 on 874 degrees of freedom
## AIC: 715.61
##
## Number of Fisher Scoring iterations: 5

Add the size of fished area

glm_1.7 <- glm(kiw_pres ~ kiw_pres_lag1*distance + area_size,
 family=binomial(link = "logit"), data=datsc_au)
summary(glm_1.7)

##
## Call:
## glm(formula = kiw_pres ~ kiw_pres_lag1 * distance + area_size,
## family = binomial(link = "logit"), data = datsc_au)
##
## Deviance Residuals:
## Min 1Q Median 3Q Max
## -2.1128 -0.5497 -0.4692 -0.3320 2.4444
##
## Coefficients:
## Estimate Std. Error z value Pr(>|z|)
## (Intercept) -2.07238 0.12387 -16.730 < 2e-16 ***
## kiw_pres_lag1 2.67479 0.20788 12.867 < 2e-16 ***
## distance -0.34647 0.16222 -2.136 0.03270 *
## area_size 0.27648 0.09393 2.944 0.00324 **
## kiw_pres_lag1:distance -0.87506 0.31217 -2.803 0.00506 **
## ---
## Signif. codes: 0 '***' 0.001 '**' 0.01 '*' 0.05 '.' 0.1 ' ' 1
##
## (Dispersion parameter for binomial family taken to be 1)
##
## Null deviance: 940.32 on 878 degrees of freedom
## Residual deviance: 709.05 on 874 degrees of freedom
## AIC: 719.05
##
## Number of Fisher Scoring iterations: 5

Add the captain

glm_1.8 <- glm(kiw_pres ~ kiw_pres_lag1*distance + captain,
 family=binomial(link = "logit"), data=datsc_au)
summary(glm_1.8)

##
## Call:
## glm(formula = kiw_pres ~ kiw_pres_lag1 * distance + captain,
## family = binomial(link = "logit"), data = datsc_au)
##
## Deviance Residuals:
## Min 1Q Median 3Q Max
## -2.1100 -0.6212 -0.3810 -0.2823 2.5670
##
## Coefficients:
## Estimate Std. Error z value Pr(>|z|)
## (Intercept) -1.6066 0.1382 -11.623 < 2e-16 ***
## kiw_pres_lag1 2.4383 0.2086 11.689 < 2e-16 ***
## distance -0.2818 0.1566 -1.800 0.07189 .
## captainPHIL -1.1477 0.2245 -5.113 3.18e-07 ***
## kiw_pres_lag1:distance -0.9578 0.3145 -3.046 0.00232 **
## ---
## Signif. codes: 0 '***' 0.001 '**' 0.01 '*' 0.05 '.' 0.1 ' ' 1
##
## (Dispersion parameter for binomial family taken to be 1)
##
## Null deviance: 940.32 on 878 degrees of freedom
## Residual deviance: 689.10 on 874 degrees of freedom
## AIC: 699.1
##
## Number of Fisher Scoring iterations: 5

### AIC summary

model.sel(list(glm_null, glm_1.1, glm_1.2, glm_1.3, glm_1.4, glm_1.5,glm_1.6,glm_1.7,glm_1.8))

## Warning in model.sel.default(list(glm_null, glm_1.1, glm_1.2, glm_1.3,
## glm_1.4, : models are not all fitted to the same data

## Model selection table
## (Int) dst kiw_prs_lg1 dst:kiw_prs_lg1 yer ssn dpt lat
## 9 -1.607 -0.2818 2.438 -0.9578
## 7 -2.089 -0.3428 2.707 -0.8763
## 8 -2.072 -0.3465 2.675 -0.8751
## 5 -2.047 -0.3025 2.607 -0.8929 0.1673
## 1 -2.043 -0.3018 2.632 -0.8376
## 4 -2.045 -0.3005 2.631 -0.8265 -0.0524
## 2 -2.041 -0.2891 2.618 -0.8282 -0.05659
## 6 -2.044 -0.3026 2.628 -0.8437
## 3 -1.897 -0.3047 2.619 -0.8598 +
## lng eff are_siz cpt df logLik AICc delta weight
## 9 + 5 -344.552 699.2 0.00 1
## 7 0.325 5 -352.806 715.7 16.51 0
## 8 0.2765 5 -354.523 719.1 19.94 0
## 5 5 -357.341 724.8 25.58 0
## 1 4 -358.875 725.8 26.62 0
## 4 5 -358.560 727.2 28.01 0
## 2 5 -358.710 727.5 28.31 0
## 6 0.05192 5 -358.730 727.5 28.36 0
## 3 7 -356.973 728.1 28.90 0
## Models ranked by AICc(x)

Model with the captain with lowest AIC

# Two terms models

glm_2.1 <- glm(kiw_pres ~ kiw_pres_lag1*distance + captain,
 family=binomial(link = "logit"), data=datsc_au)
summary(glm_2.1)

##
## Call:
## glm(formula = kiw_pres ~ kiw_pres_lag1 * distance + captain,
## family = binomial(link = "logit"), data = datsc_au)
##
## Deviance Residuals:
## Min 1Q Median 3Q Max
## -2.1100 -0.6212 -0.3810 -0.2823 2.5670
##
## Coefficients:
## Estimate Std. Error z value Pr(>|z|)
## (Intercept) -1.6066 0.1382 -11.623 < 2e-16 ***
## kiw_pres_lag1 2.4383 0.2086 11.689 < 2e-16 ***
## distance -0.2818 0.1566 -1.800 0.07189 .
## captainPHIL -1.1477 0.2245 -5.113 3.18e-07 ***
## kiw_pres_lag1:distance -0.9578 0.3145 -3.046 0.00232 **
## ---
## Signif. codes: 0 '***' 0.001 '**' 0.01 '*' 0.05 '.' 0.1 ' ' 1
##
## (Dispersion parameter for binomial family taken to be 1)
##
## Null deviance: 940.32 on 878 degrees of freedom
## Residual deviance: 689.10 on 874 degrees of freedom
## AIC: 699.1
##
## Number of Fisher Scoring iterations: 5

Model with the year

glm_2.2 <- glm(kiw_pres ~ kiw_pres_lag1*distance + captain + year,
 family=binomial(link = "logit"), data=datsc_au)
summary(glm_2.2)

##
## Call:
## glm(formula = kiw_pres ~ kiw_pres_lag1 * distance + captain +
## year, family = binomial(link = "logit"), data = datsc_au)
##
## Deviance Residuals:
## Min 1Q Median 3Q Max
## -2.1087 -0.6203 -0.3797 -0.2844 2.5707
##
## Coefficients:
## Estimate Std. Error z value Pr(>|z|)
## (Intercept) -1.60676 0.13823 -11.624 < 2e-16 ***
## kiw_pres_lag1 2.43397 0.20959 11.613 < 2e-16 ***
## distance -0.27687 0.15821 -1.750 0.0801 .
## captainPHIL -1.14470 0.22495 -5.089 3.6e-07 ***
## year -0.02046 0.09961 -0.205 0.8372
## kiw_pres_lag1:distance -0.95469 0.31449 -3.036 0.0024 **
## ---
## Signif. codes: 0 '***' 0.001 '**' 0.01 '*' 0.05 '.' 0.1 ' ' 1
##
## (Dispersion parameter for binomial family taken to be 1)
##
## Null deviance: 940.32 on 878 degrees of freedom
## Residual deviance: 689.06 on 873 degrees of freedom
## AIC: 701.06
##
## Number of Fisher Scoring iterations: 5

Model with the season

glm_2.3 <- glm(kiw_pres ~ kiw_pres_lag1*distance + captain + season,
 family=binomial(link = "logit"), data=datsc_au)
summary(glm_2.3)

##
## Call:
## glm(formula = kiw_pres ~ kiw_pres_lag1 * distance + captain +
## season, family = binomial(link = "logit"), data = datsc_au)
##
## Deviance Residuals:
## Min 1Q Median 3Q Max
## -2.2136 -0.5955 -0.3928 -0.2653 2.6336
##
## Coefficients:
## Estimate Std. Error z value Pr(>|z|)
## (Intercept) -1.38563 0.19653 -7.051 1.78e-12 ***
## kiw_pres_lag1 2.43019 0.21002 11.571 < 2e-16 ***
## distance -0.28648 0.15762 -1.818 0.06913 .
## captainPHIL -1.17078 0.22621 -5.176 2.27e-07 ***
## season2_Autumn -0.09732 0.25073 -0.388 0.69792
## season3_Winter -0.44996 0.38649 -1.164 0.24434
## season4_Spring -0.46787 0.24503 -1.909 0.05621 .
## kiw_pres_lag1:distance -0.98713 0.31676 -3.116 0.00183 **
## ---
## Signif. codes: 0 '***' 0.001 '**' 0.01 '*' 0.05 '.' 0.1 ' ' 1
##
## (Dispersion parameter for binomial family taken to be 1)
##
## Null deviance: 940.32 on 878 degrees of freedom
## Residual deviance: 684.55 on 871 degrees of freedom
## AIC: 700.55
##
## Number of Fisher Scoring iterations: 5

Model with the depth

glm_2.4 <- glm(kiw_pres ~ kiw_pres_lag1*distance + captain + depth,
 family=binomial(link = "logit"), data=datsc_au)
summary(glm_2.4)

##
## Call:
## glm(formula = kiw_pres ~ kiw_pres_lag1 * distance + captain +
## depth, family = binomial(link = "logit"), data = datsc_au)
##
## Deviance Residuals:
## Min 1Q Median 3Q Max
## -2.1150 -0.6224 -0.3798 -0.2834 2.5656
##
## Coefficients:
## Estimate Std. Error z value Pr(>|z|)
## (Intercept) -1.6093 0.1383 -11.633 < 2e-16 ***
## kiw_pres_lag1 2.4397 0.2090 11.671 < 2e-16 ***
## distance -0.2822 0.1564 -1.805 0.07114 .
## captainPHIL -1.1451 0.2244 -5.102 3.36e-07 ***
## depth -0.0541 0.1050 -0.515 0.60635
## kiw_pres_lag1:distance -0.9473 0.3142 -3.015 0.00257 **
## ---
## Signif. codes: 0 '***' 0.001 '**' 0.01 '*' 0.05 '.' 0.1 ' ' 1
##
## (Dispersion parameter for binomial family taken to be 1)
##
## Null deviance: 937.35 on 877 degrees of freedom
## Residual deviance: 688.59 on 872 degrees of freedom
## (1 observation deleted due to missingness)
## AIC: 700.59
##
## Number of Fisher Scoring iterations: 5

Model with the longitude

glm_2.5 <- glm(kiw_pres ~ kiw_pres_lag1*distance + captain + long,
 family=binomial(link = "logit"), data=datsc_au)
summary(glm_2.5)

##
## Call:
## glm(formula = kiw_pres ~ kiw_pres_lag1 * distance + captain +
## long, family = binomial(link = "logit"), data = datsc_au)
##
## Deviance Residuals:
## Min 1Q Median 3Q Max
## -2.1224 -0.6193 -0.3805 -0.2818 2.5554
##
## Coefficients:
## Estimate Std. Error z value Pr(>|z|)
## (Intercept) -1.60684 0.13832 -11.617 < 2e-16 ***
## kiw_pres_lag1 2.43634 0.20881 11.668 < 2e-16 ***
## distance -0.28198 0.15667 -1.800 0.07189 .
## captainPHIL -1.14411 0.22477 -5.090 3.58e-07 ***
## long 0.02451 0.09980 0.246 0.80600
## kiw_pres_lag1:distance -0.96191 0.31524 -3.051 0.00228 **
## ---
## Signif. codes: 0 '***' 0.001 '**' 0.01 '*' 0.05 '.' 0.1 ' ' 1
##
## (Dispersion parameter for binomial family taken to be 1)
##
## Null deviance: 940.32 on 878 degrees of freedom
## Residual deviance: 689.04 on 873 degrees of freedom
## AIC: 701.04
##
## Number of Fisher Scoring iterations: 5

Model with the total effort

glm_2.6 <- glm(kiw_pres ~ kiw_pres_lag1*distance + captain + effort,
 family=binomial(link = "logit"), data=datsc_au)
summary(glm_2.6)

##
## Call:
## glm(formula = kiw_pres ~ kiw_pres_lag1 * distance + captain +
## effort, family = binomial(link = "logit"), data = datsc_au)
##
## Deviance Residuals:
## Min 1Q Median 3Q Max
## -2.3156 -0.5954 -0.3724 -0.2389 2.6716
##
## Coefficients:
## Estimate Std. Error z value Pr(>|z|)
## (Intercept) -1.64373 0.14108 -11.651 < 2e-16 ***
## kiw_pres_lag1 2.51524 0.21437 11.733 < 2e-16 ***
## distance -0.32257 0.16200 -1.991 0.046455 *
## captainPHIL -1.18180 0.22713 -5.203 1.96e-07 ***
## effort 0.35255 0.09649 3.654 0.000258 ***
## kiw_pres_lag1:distance -1.00374 0.31966 -3.140 0.001689 **
## ---
## Signif. codes: 0 '***' 0.001 '**' 0.01 '*' 0.05 '.' 0.1 ' ' 1
##
## (Dispersion parameter for binomial family taken to be 1)
##
## Null deviance: 940.32 on 878 degrees of freedom
## Residual deviance: 675.83 on 873 degrees of freedom
## AIC: 687.83
##
## Number of Fisher Scoring iterations: 5

Model with the size of fished area

glm_2.7 <- glm(kiw_pres ~ kiw_pres_lag1*distance + captain + area_size,
 family=binomial(link = "logit"), data=datsc_au)
summary(glm_2.7)

##
## Call:
## glm(formula = kiw_pres ~ kiw_pres_lag1 * distance + captain +
## area_size, family = binomial(link = "logit"), data = datsc_au)
##
## Deviance Residuals:
## Min 1Q Median 3Q Max
## -2.2962 -0.5911 -0.3787 -0.2405 2.6287
##
## Coefficients:
## Estimate Std. Error z value Pr(>|z|)
## (Intercept) -1.62350 0.13947 -11.640 < 2e-16 ***
## kiw_pres_lag1 2.47968 0.21209 11.692 < 2e-16 ***
## distance -0.33026 0.16202 -2.038 0.041508 *
## captainPHIL -1.21872 0.22954 -5.310 1.1e-07 ***
## area_size 0.32310 0.09673 3.340 0.000837 ***
## kiw_pres_lag1:distance -0.99930 0.31944 -3.128 0.001758 **
## ---
## Signif. codes: 0 '***' 0.001 '**' 0.01 '*' 0.05 '.' 0.1 ' ' 1
##
## (Dispersion parameter for binomial family taken to be 1)
##
## Null deviance: 940.32 on 878 degrees of freedom
## Residual deviance: 677.80 on 873 degrees of freedom
## AIC: 689.8
##
## Number of Fisher Scoring iterations: 5

Model with the latitude

glm_2.8 <- glm(kiw_pres ~ kiw_pres_lag1*distance + captain + lat,
 family=binomial(link = "logit"), data=datsc_au)
summary(glm_2.8)

##
## Call:
## glm(formula = kiw_pres ~ kiw_pres_lag1 * distance + captain +
## lat, family = binomial(link = "logit"), data = datsc_au)
##
## Deviance Residuals:
## Min 1Q Median 3Q Max
## -2.1005 -0.6126 -0.3866 -0.2826 2.5727
##
## Coefficients:
## Estimate Std. Error z value Pr(>|z|)
## (Intercept) -1.61748 0.13918 -11.622 < 2e-16 ***
## kiw_pres_lag1 2.42477 0.20912 11.595 < 2e-16 ***
## distance -0.28099 0.15711 -1.788 0.07370 .
## captainPHIL -1.11779 0.22575 -4.951 7.37e-07 ***
## lat 0.10573 0.09751 1.084 0.27824
## kiw_pres_lag1:distance -0.99320 0.31872 -3.116 0.00183 **
## ---
## Signif. codes: 0 '***' 0.001 '**' 0.01 '*' 0.05 '.' 0.1 ' ' 1
##
## (Dispersion parameter for binomial family taken to be 1)
##
## Null deviance: 940.32 on 878 degrees of freedom
## Residual deviance: 687.93 on 873 degrees of freedom
## AIC: 699.93
##
## Number of Fisher Scoring iterations: 5

### AIC summary

model.sel(list(glm_2.1, glm_2.2, glm_2.3, glm_2.4, glm_2.5,glm_2.6,glm_2.7,glm_2.8))

## Warning in model.sel.default(list(glm_2.1, glm_2.2, glm_2.3, glm_2.4,
## glm_2.5, : models are not all fitted to the same data

## Model selection table
## (Int) cpt dst kiw_prs_lg1 dst:kiw_prs_lg1 yer ssn dpt
## 6 -1.644 + -0.3226 2.515 -1.0040
## 7 -1.623 + -0.3303 2.480 -0.9993
## 1 -1.607 + -0.2818 2.438 -0.9578
## 8 -1.617 + -0.2810 2.425 -0.9932
## 4 -1.609 + -0.2822 2.440 -0.9473 -0.0541
## 3 -1.386 + -0.2865 2.430 -0.9871 +
## 5 -1.607 + -0.2820 2.436 -0.9619
## 2 -1.607 + -0.2769 2.434 -0.9547 -0.02046
## lng eff are_siz lat df logLik AICc delta weight
## 6 0.3526 6 -337.917 687.9 0.00 0.722
## 7 0.3231 6 -338.902 689.9 1.97 0.270
## 1 5 -344.552 699.2 11.24 0.003
## 8 0.1057 6 -343.963 700.0 12.09 0.002
## 4 6 -344.296 700.7 12.76 0.001
## 3 8 -342.277 700.7 12.79 0.001
## 5 0.02451 6 -344.522 701.1 13.21 0.001
## 2 6 -344.531 701.2 13.23 0.001
## Models ranked by AICc(x)

Model with the effort with lowest AIC

# Three terms models

glm_3.1 <- glm(kiw_pres ~ kiw_pres_lag1*distance + captain+effort,
 family=binomial(link = "logit"), data=datsc_au)
summary(glm_3.1)

##
## Call:
## glm(formula = kiw_pres ~ kiw_pres_lag1 * distance + captain +
## effort, family = binomial(link = "logit"), data = datsc_au)
##
## Deviance Residuals:
## Min 1Q Median 3Q Max
## -2.3156 -0.5954 -0.3724 -0.2389 2.6716
##
## Coefficients:
## Estimate Std. Error z value Pr(>|z|)
## (Intercept) -1.64373 0.14108 -11.651 < 2e-16 ***
## kiw_pres_lag1 2.51524 0.21437 11.733 < 2e-16 ***
## distance -0.32257 0.16200 -1.991 0.046455 *
## captainPHIL -1.18180 0.22713 -5.203 1.96e-07 ***
## effort 0.35255 0.09649 3.654 0.000258 ***
## kiw_pres_lag1:distance -1.00374 0.31966 -3.140 0.001689 **
## ---
## Signif. codes: 0 '***' 0.001 '**' 0.01 '*' 0.05 '.' 0.1 ' ' 1
##
## (Dispersion parameter for binomial family taken to be 1)
##
## Null deviance: 940.32 on 878 degrees of freedom
## Residual deviance: 675.83 on 873 degrees of freedom
## AIC: 687.83
##
## Number of Fisher Scoring iterations: 5

Model with the season

glm_3.2 <- glm(kiw_pres ~ kiw_pres_lag1*distance + captain+effort+ season,
 family=binomial(link = "logit"), data=datsc_au)
summary(glm_3.2)

##
## Call:
## glm(formula = kiw_pres ~ kiw_pres_lag1 * distance + captain +
## effort + season, family = binomial(link = "logit"), data = datsc_au)
##
## Deviance Residuals:
## Min 1Q Median 3Q Max
## -2.2122 -0.5734 -0.3840 -0.2209 2.6519
##
## Coefficients:
## Estimate Std. Error z value Pr(>|z|)
## (Intercept) -1.37235 0.19831 -6.920 4.51e-12 ***
## kiw_pres_lag1 2.51863 0.21687 11.613 < 2e-16 ***
## distance -0.32976 0.16354 -2.016 0.043763 *
## captainPHIL -1.21255 0.22939 -5.286 1.25e-07 ***
## effort 0.38079 0.09917 3.840 0.000123 ***
## season2_Autumn -0.16283 0.25565 -0.637 0.524180
## season3_Winter -0.62747 0.39094 -1.605 0.108488
## season4_Spring -0.53156 0.24745 -2.148 0.031705 *
## kiw_pres_lag1:distance -1.04275 0.32221 -3.236 0.001211 **
## ---
## Signif. codes: 0 '***' 0.001 '**' 0.01 '*' 0.05 '.' 0.1 ' ' 1
##
## (Dispersion parameter for binomial family taken to be 1)
##
## Null deviance: 940.32 on 878 degrees of freedom
## Residual deviance: 669.81 on 870 degrees of freedom
## AIC: 687.81
##
## Number of Fisher Scoring iterations: 5

Model with the depth

glm_3.3 <- glm(kiw_pres ~ kiw_pres_lag1*distance +captain+effort+depth,
 family=binomial(link = "logit"), data=datsc_au)
summary(glm_3.3)

##
## Call:
## glm(formula = kiw_pres ~ kiw_pres_lag1 * distance + captain +
## effort + depth, family = binomial(link = "logit"), data = datsc_au)
##
## Deviance Residuals:
## Min 1Q Median 3Q Max
## -2.3340 -0.5931 -0.3764 -0.2339 2.6703
##
## Coefficients:
## Estimate Std. Error z value Pr(>|z|)
## (Intercept) -1.64849 0.14127 -11.670 < 2e-16 ***
## kiw_pres_lag1 2.51948 0.21517 11.710 < 2e-16 ***
## distance -0.32241 0.16171 -1.994 0.046179 *
## captainPHIL -1.17757 0.22708 -5.186 2.15e-07 ***
## effort 0.36145 0.09728 3.715 0.000203 ***
## depth -0.08444 0.10967 -0.770 0.441335
## kiw_pres_lag1:distance -0.99000 0.31955 -3.098 0.001947 **
## ---
## Signif. codes: 0 '***' 0.001 '**' 0.01 '*' 0.05 '.' 0.1 ' ' 1
##
## (Dispersion parameter for binomial family taken to be 1)
##
## Null deviance: 937.35 on 877 degrees of freedom
## Residual deviance: 674.83 on 871 degrees of freedom
## (1 observation deleted due to missingness)
## AIC: 688.83
##
## Number of Fisher Scoring iterations: 5

Model with the longitude

glm_3.4 <- glm(kiw_pres ~ kiw_pres_lag1*distance + captain+effort+long,
 family=binomial(link = "logit"), data=datsc_au)
summary(glm_3.4)

##
## Call:
## glm(formula = kiw_pres ~ kiw_pres_lag1 * distance + captain +
## effort + long, family = binomial(link = "logit"), data = datsc_au)
##
## Deviance Residuals:
## Min 1Q Median 3Q Max
## -2.3060 -0.5946 -0.3732 -0.2389 2.6653
##
## Coefficients:
## Estimate Std. Error z value Pr(>|z|)
## (Intercept) -1.64388 0.14114 -11.647 < 2e-16 ***
## kiw_pres_lag1 2.51406 0.21457 11.717 < 2e-16 ***
## distance -0.32266 0.16204 -1.991 0.046462 *
## captainPHIL -1.17977 0.22753 -5.185 2.16e-07 ***
## effort 0.35199 0.09654 3.646 0.000266 ***
## long 0.01347 0.10100 0.133 0.893932
## kiw_pres_lag1:distance -1.00555 0.32006 -3.142 0.001679 **
## ---
## Signif. codes: 0 '***' 0.001 '**' 0.01 '*' 0.05 '.' 0.1 ' ' 1
##
## (Dispersion parameter for binomial family taken to be 1)
##
## Null deviance: 940.32 on 878 degrees of freedom
## Residual deviance: 675.82 on 872 degrees of freedom
## AIC: 689.82
##
## Number of Fisher Scoring iterations: 5

Model with the latitude

glm_3.5 <- glm(kiw_pres ~ kiw_pres_lag1*distance + captain+effort+lat,
 family=binomial(link = "logit"), data=datsc_au)
summary(glm_3.5)

##
## Call:
## glm(formula = kiw_pres ~ kiw_pres_lag1 * distance + captain +
## effort + lat, family = binomial(link = "logit"), data = datsc_au)
##
## Deviance Residuals:
## Min 1Q Median 3Q Max
## -2.2538 -0.5842 -0.3758 -0.2422 2.6466
##
## Coefficients:
## Estimate Std. Error z value Pr(>|z|)
## (Intercept) -1.65395 0.14207 -11.642 < 2e-16 ***
## kiw_pres_lag1 2.50370 0.21480 11.656 < 2e-16 ***
## distance -0.32296 0.16259 -1.986 0.046998 *
## captainPHIL -1.15617 0.22835 -5.063 4.12e-07 ***
## effort 0.34872 0.09636 3.619 0.000296 ***
## lat 0.09495 0.09839 0.965 0.334528
## kiw_pres_lag1:distance -1.03187 0.32244 -3.200 0.001373 **
## ---
## Signif. codes: 0 '***' 0.001 '**' 0.01 '*' 0.05 '.' 0.1 ' ' 1
##
## (Dispersion parameter for binomial family taken to be 1)
##
## Null deviance: 940.32 on 878 degrees of freedom
## Residual deviance: 674.90 on 872 degrees of freedom
## AIC: 688.9
##
## Number of Fisher Scoring iterations: 5

Model with the size of area fished

glm_3.6 <- glm(kiw_pres ~ kiw_pres_lag1*distance + captain+effort+area_size,
 family=binomial(link = "logit"), data=datsc_au)
summary(glm_3.6)

##
## Call:
## glm(formula = kiw_pres ~ kiw_pres_lag1 * distance + captain +
## effort + area_size, family = binomial(link = "logit"), data = datsc_au)
##
## Deviance Residuals:
## Min 1Q Median 3Q Max
## -2.3781 -0.5914 -0.3756 -0.2345 2.6697
##
## Coefficients:
## Estimate Std. Error z value Pr(>|z|)
## (Intercept) -1.6395 0.1408 -11.641 < 2e-16 ***
## kiw_pres_lag1 2.5131 0.2146 11.711 < 2e-16 ***
## distance -0.3337 0.1631 -2.045 0.0408 *
## captainPHIL -1.2076 0.2293 -5.266 1.39e-07 ***
## effort 0.2448 0.1310 1.868 0.0618 .
## area_size 0.1591 0.1304 1.220 0.2226
## kiw_pres_lag1:distance -1.0123 0.3208 -3.156 0.0016 **
## ---
## Signif. codes: 0 '***' 0.001 '**' 0.01 '*' 0.05 '.' 0.1 ' ' 1
##
## (Dispersion parameter for binomial family taken to be 1)
##
## Null deviance: 940.32 on 878 degrees of freedom
## Residual deviance: 674.34 on 872 degrees of freedom
## AIC: 688.34
##
## Number of Fisher Scoring iterations: 5

Model with the year

glm_3.7 <- glm(kiw_pres ~ kiw_pres_lag1*distance + captain+effort+year,
 family=binomial(link = "logit"), data=datsc_au)
summary(glm_3.7)

##
## Call:
## glm(formula = kiw_pres ~ kiw_pres_lag1 * distance + captain +
## effort + year, family = binomial(link = "logit"), data = datsc_au)
##
## Deviance Residuals:
## Min 1Q Median 3Q Max
## -2.3061 -0.5917 -0.3737 -0.2377 2.6667
##
## Coefficients:
## Estimate Std. Error z value Pr(>|z|)
## (Intercept) -1.6444 0.1411 -11.652 < 2e-16 ***
## kiw_pres_lag1 2.5261 0.2163 11.679 < 2e-16 ***
## distance -0.3339 0.1648 -2.026 0.042801 *
## captainPHIL -1.1892 0.2280 -5.216 1.83e-07 ***
## effort 0.3596 0.0981 3.666 0.000246 ***
## year 0.0421 0.1027 0.410 0.681899
## kiw_pres_lag1:distance -1.0116 0.3208 -3.153 0.001617 **
## ---
## Signif. codes: 0 '***' 0.001 '**' 0.01 '*' 0.05 '.' 0.1 ' ' 1
##
## (Dispersion parameter for binomial family taken to be 1)
##
## Null deviance: 940.32 on 878 degrees of freedom
## Residual deviance: 675.67 on 872 degrees of freedom
## AIC: 689.67
##
## Number of Fisher Scoring iterations: 5

### AIC summary

model.sel(list(glm_3.1, glm_3.2, glm_3.3, glm_3.4, glm_3.5,glm_3.6,glm_3.7))

## Warning in model.sel.default(list(glm_3.1, glm_3.2, glm_3.3, glm_3.4,
## glm_3.5, : models are not all fitted to the same data

## Model selection table
## (Int) cpt dst eff kiw_prs_lg1 dst:kiw_prs_lg1 ssn dpt
## 1 -1.644 + -0.3226 0.3526 2.515 -1.004
## 2 -1.372 + -0.3298 0.3808 2.519 -1.043 +
## 6 -1.639 + -0.3337 0.2448 2.513 -1.012
## 3 -1.648 + -0.3224 0.3614 2.519 -0.990 -0.08444
## 5 -1.654 + -0.3230 0.3487 2.504 -1.032
## 7 -1.644 + -0.3339 0.3596 2.526 -1.012
## 4 -1.644 + -0.3227 0.3520 2.514 -1.006
## lng lat are_siz yer df logLik AICc delta weight
## 1 6 -337.917 687.9 0.00 0.215
## 2 9 -334.906 688.0 0.09 0.206
## 6 0.1591 7 -337.171 688.5 0.54 0.164
## 3 7 -337.415 689.0 1.03 0.128
## 5 0.09495 7 -337.450 689.0 1.10 0.124
## 7 0.0421 7 -337.833 689.8 1.86 0.085
## 4 0.01347 7 -337.908 689.9 2.01 0.078
## Models ranked by AICc(x)

Model with the season with lowest AIC

# Four terms models

glm_4.1 <- glm(kiw_pres ~ kiw_pres_lag1*distance + captain+effort+season,
 family=binomial(link = "logit"), data=datsc_au)
summary(glm_4.1)

##
## Call:
## glm(formula = kiw_pres ~ kiw_pres_lag1 * distance + captain +
## effort + season, family = binomial(link = "logit"), data = datsc_au)
##
## Deviance Residuals:
## Min 1Q Median 3Q Max
## -2.2122 -0.5734 -0.3840 -0.2209 2.6519
##
## Coefficients:
## Estimate Std. Error z value Pr(>|z|)
## (Intercept) -1.37235 0.19831 -6.920 4.51e-12 ***
## kiw_pres_lag1 2.51863 0.21687 11.613 < 2e-16 ***
## distance -0.32976 0.16354 -2.016 0.043763 *
## captainPHIL -1.21255 0.22939 -5.286 1.25e-07 ***
## effort 0.38079 0.09917 3.840 0.000123 ***
## season2_Autumn -0.16283 0.25565 -0.637 0.524180
## season3_Winter -0.62747 0.39094 -1.605 0.108488
## season4_Spring -0.53156 0.24745 -2.148 0.031705 *
## kiw_pres_lag1:distance -1.04275 0.32221 -3.236 0.001211 **
## ---
## Signif. codes: 0 '***' 0.001 '**' 0.01 '*' 0.05 '.' 0.1 ' ' 1
##
## (Dispersion parameter for binomial family taken to be 1)
##
## Null deviance: 940.32 on 878 degrees of freedom
## Residual deviance: 669.81 on 870 degrees of freedom
## AIC: 687.81
##
## Number of Fisher Scoring iterations: 5

Model with the year

glm_4.2 <- glm(kiw_pres ~ kiw_pres_lag1*distance + captain+effort+season+year,
 family=binomial(link = "logit"), data=datsc_au)
summary(glm_4.2)

##
## Call:
## glm(formula = kiw_pres ~ kiw_pres_lag1 * distance + captain +
## effort + season + year, family = binomial(link = "logit"),
## data = datsc_au)
##
## Deviance Residuals:
## Min 1Q Median 3Q Max
## -2.2117 -0.5730 -0.3843 -0.2201 2.6504
##
## Coefficients:
## Estimate Std. Error z value Pr(>|z|)
## (Intercept) -1.37407 0.19907 -6.902 5.12e-12 ***
## kiw_pres_lag1 2.52116 0.21829 11.549 < 2e-16 ***
## distance -0.33266 0.16598 -2.004 0.045046 *
## captainPHIL -1.21422 0.22997 -5.280 1.29e-07 ***
## effort 0.38239 0.10035 3.811 0.000139 ***
## season2_Autumn -0.16114 0.25617 -0.629 0.529343
## season3_Winter -0.61993 0.39748 -1.560 0.118839
## season4_Spring -0.52989 0.24797 -2.137 0.032606 *
## year 0.01095 0.10458 0.105 0.916640
## kiw_pres_lag1:distance -1.04461 0.32288 -3.235 0.001215 **
## ---
## Signif. codes: 0 '***' 0.001 '**' 0.01 '*' 0.05 '.' 0.1 ' ' 1
##
## (Dispersion parameter for binomial family taken to be 1)
##
## Null deviance: 940.32 on 878 degrees of freedom
## Residual deviance: 669.80 on 869 degrees of freedom
## AIC: 689.8
##
## Number of Fisher Scoring iterations: 5

Model with the depth

glm_4.3 <- glm(kiw_pres ~ kiw_pres_lag1*distance + captain+effort+season+depth,
 family=binomial(link = "logit"), data=datsc_au)
summary(glm_4.3)

##
## Call:
## glm(formula = kiw_pres ~ kiw_pres_lag1 * distance + captain +
## effort + season + depth, family = binomial(link = "logit"),
## data = datsc_au)
##
## Deviance Residuals:
## Min 1Q Median 3Q Max
## -2.2153 -0.5749 -0.3853 -0.2187 2.6514
##
## Coefficients:
## Estimate Std. Error z value Pr(>|z|)
## (Intercept) -1.37953 0.20075 -6.872 6.34e-12 ***
## kiw_pres_lag1 2.51580 0.21748 11.568 < 2e-16 ***
## distance -0.32999 0.16348 -2.018 0.043539 *
## captainPHIL -1.20823 0.22927 -5.270 1.36e-07 ***
## effort 0.38566 0.09961 3.872 0.000108 ***
## season2_Autumn -0.15847 0.25602 -0.619 0.535927
## season3_Winter -0.62083 0.39124 -1.587 0.112551
## season4_Spring -0.52435 0.25514 -2.055 0.039865 *
## depth -0.02712 0.11331 -0.239 0.810817
## kiw_pres_lag1:distance -1.02493 0.32251 -3.178 0.001483 **
## ---
## Signif. codes: 0 '***' 0.001 '**' 0.01 '*' 0.05 '.' 0.1 ' ' 1
##
## (Dispersion parameter for binomial family taken to be 1)
##
## Null deviance: 937.35 on 877 degrees of freedom
## Residual deviance: 669.23 on 868 degrees of freedom
## (1 observation deleted due to missingness)
## AIC: 689.23
##
## Number of Fisher Scoring iterations: 5

Model with the longitude

glm_4.4 <- glm(kiw_pres ~ kiw_pres_lag1*distance + captain+effort+season+long,
 family=binomial(link = "logit"), data=datsc_au)
summary(glm_4.4)

##
## Call:
## glm(formula = kiw_pres ~ kiw_pres_lag1 * distance + captain +
## effort + season + long, family = binomial(link = "logit"),
## data = datsc_au)
##
## Deviance Residuals:
## Min 1Q Median 3Q Max
## -2.2242 -0.5723 -0.3837 -0.2217 2.6553
##
## Coefficients:
## Estimate Std. Error z value Pr(>|z|)
## (Intercept) -1.37430 0.19883 -6.912 4.78e-12 ***
## kiw_pres_lag1 2.51915 0.21690 11.614 < 2e-16 ***
## distance -0.32952 0.16350 -2.015 0.043868 *
## captainPHIL -1.21388 0.22971 -5.284 1.26e-07 ***
## effort 0.38120 0.09927 3.840 0.000123 ***
## season2_Autumn -0.15310 0.26664 -0.574 0.565841
## season3_Winter -0.61986 0.39508 -1.569 0.116663
## season4_Spring -0.53502 0.24898 -2.149 0.031646 *
## long -0.01403 0.10957 -0.128 0.898076
## kiw_pres_lag1:distance -1.04092 0.32240 -3.229 0.001244 **
## ---
## Signif. codes: 0 '***' 0.001 '**' 0.01 '*' 0.05 '.' 0.1 ' ' 1
##
## (Dispersion parameter for binomial family taken to be 1)
##
## Null deviance: 940.32 on 878 degrees of freedom
## Residual deviance: 669.79 on 869 degrees of freedom
## AIC: 689.79
##
## Number of Fisher Scoring iterations: 5

Model with the latitude

glm_4.5 <- glm(kiw_pres ~ kiw_pres_lag1*distance + captain+effort+season+lat,
 family=binomial(link = "logit"), data=datsc_au)
summary(glm_4.5)

##
## Call:
## glm(formula = kiw_pres ~ kiw_pres_lag1 * distance + captain +
## effort + season + lat, family = binomial(link = "logit"),
## data = datsc_au)
##
## Deviance Residuals:
## Min 1Q Median 3Q Max
## -2.1898 -0.5770 -0.3797 -0.2178 2.6468
##
## Coefficients:
## Estimate Std. Error z value Pr(>|z|)
## (Intercept) -1.37158 0.19896 -6.894 5.43e-12 ***
## kiw_pres_lag1 2.51060 0.21726 11.556 < 2e-16 ***
## distance -0.32919 0.16386 -2.009 0.044535 *
## captainPHIL -1.19561 0.23088 -5.179 2.24e-07 ***
## effort 0.37717 0.09911 3.806 0.000141 ***
## season2_Autumn -0.20411 0.26513 -0.770 0.441391
## season3_Winter -0.60263 0.39310 -1.533 0.125272
## season4_Spring -0.52435 0.24759 -2.118 0.034193 *
## lat 0.06388 0.10487 0.609 0.542459
## kiw_pres_lag1:distance -1.06138 0.32477 -3.268 0.001083 **
## ---
## Signif. codes: 0 '***' 0.001 '**' 0.01 '*' 0.05 '.' 0.1 ' ' 1
##
## (Dispersion parameter for binomial family taken to be 1)
##
## Null deviance: 940.32 on 878 degrees of freedom
## Residual deviance: 669.44 on 869 degrees of freedom
## AIC: 689.44
##
## Number of Fisher Scoring iterations: 5

Model with the size of area fished

glm_4.6 <- glm(kiw_pres ~ kiw_pres_lag1*distance + captain+effort+season+area_size,
 family=binomial(link = "logit"), data=datsc_au)
summary(glm_4.6)

##
## Call:
## glm(formula = kiw_pres ~ kiw_pres_lag1 * distance + captain +
## effort + season + area_size, family = binomial(link = "logit"),
## data = datsc_au)
##
## Deviance Residuals:
## Min 1Q Median 3Q Max
## -2.2750 -0.5767 -0.3846 -0.2162 2.6500
##
## Coefficients:
## Estimate Std. Error z value Pr(>|z|)
## (Intercept) -1.3775 0.1984 -6.943 3.84e-12 ***
## kiw_pres_lag1 2.5156 0.2170 11.595 < 2e-16 ***
## distance -0.3398 0.1646 -2.065 0.03897 *
## captainPHIL -1.2341 0.2314 -5.334 9.59e-08 ***
## effort 0.2846 0.1346 2.114 0.03447 *
## season2_Autumn -0.1545 0.2558 -0.604 0.54598
## season3_Winter -0.5949 0.3932 -1.513 0.13028
## season4_Spring -0.5201 0.2476 -2.101 0.03567 *
## area_size 0.1391 0.1314 1.059 0.28980
## kiw_pres_lag1:distance -1.0475 0.3229 -3.244 0.00118 **
## ---
## Signif. codes: 0 '***' 0.001 '**' 0.01 '*' 0.05 '.' 0.1 ' ' 1
##
## (Dispersion parameter for binomial family taken to be 1)
##
## Null deviance: 940.32 on 878 degrees of freedom
## Residual deviance: 668.69 on 869 degrees of freedom
## AIC: 688.69
##
## Number of Fisher Scoring iterations: 5

### AIC summary

model.sel(list(glm_4.1, glm_4.2, glm_4.3, glm_4.4, glm_4.5,glm_4.6))

## Warning in model.sel.default(list(glm_4.1, glm_4.2, glm_4.3, glm_4.4,
## glm_4.5, : models are not all fitted to the same data

## Model selection table
## (Int) cpt dst eff kiw_prs_lg1 ssn dst:kiw_prs_lg1 yer
## 1 -1.372 + -0.3298 0.3808 2.519 + -1.043
## 6 -1.377 + -0.3398 0.2846 2.516 + -1.047
## 3 -1.380 + -0.3300 0.3857 2.516 + -1.025
## 5 -1.372 + -0.3292 0.3772 2.511 + -1.061
## 4 -1.374 + -0.3295 0.3812 2.519 + -1.041
## 2 -1.374 + -0.3327 0.3824 2.521 + -1.045 0.01095
## dpt lng lat are_siz df logLik AICc delta weight
## 1 9 -334.906 688.0 0.00 0.306
## 6 0.1391 10 -334.344 688.9 0.92 0.193
## 3 -0.02712 10 -334.615 689.5 1.47 0.147
## 5 0.06388 10 -334.720 689.7 1.68 0.132
## 4 -0.01403 10 -334.897 690.0 2.03 0.111
## 2 10 -334.900 690.1 2.04 0.111
## Models ranked by AICc(x)

None of the 4 terms models further improved the AIC

the final model should be

glm_4.1 <- glm(kiw_pres ~ kiw_pres_lag1*distance + captain+effort+season,
 family=binomial(link = "logit"), data=datsc_au)
summary(glm_4.1)

##
## Call:
## glm(formula = kiw_pres ~ kiw_pres_lag1 * distance + captain +
## effort + season, family = binomial(link = "logit"), data = datsc_au)
##
## Deviance Residuals:
## Min 1Q Median 3Q Max
## -2.2122 -0.5734 -0.3840 -0.2209 2.6519
##
## Coefficients:
## Estimate Std. Error z value Pr(>|z|)
## (Intercept) -1.37235 0.19831 -6.920 4.51e-12 ***
## kiw_pres_lag1 2.51863 0.21687 11.613 < 2e-16 ***
## distance -0.32976 0.16354 -2.016 0.043763 *
## captainPHIL -1.21255 0.22939 -5.286 1.25e-07 ***
## effort 0.38079 0.09917 3.840 0.000123 ***
## season2_Autumn -0.16283 0.25565 -0.637 0.524180
## season3_Winter -0.62747 0.39094 -1.605 0.108488
## season4_Spring -0.53156 0.24745 -2.148 0.031705 *
## kiw_pres_lag1:distance -1.04275 0.32221 -3.236 0.001211 **
## ---
## Signif. codes: 0 '***' 0.001 '**' 0.01 '*' 0.05 '.' 0.1 ' ' 1
##
## (Dispersion parameter for binomial family taken to be 1)
##
## Null deviance: 940.32 on 878 degrees of freedom
## Residual deviance: 669.81 on 870 degrees of freedom
## AIC: 687.81
##
## Number of Fisher Scoring iterations: 5

# Model validation

plots of residuals versus fitted values for check of linearity and homoscedasticity of variance

plot(fitted(glm_4.1), resid(glm_4.1), main="Residual Plot for linearity and homoscedasticity of variance",xlab="Fitted value",ylab="Residuals")


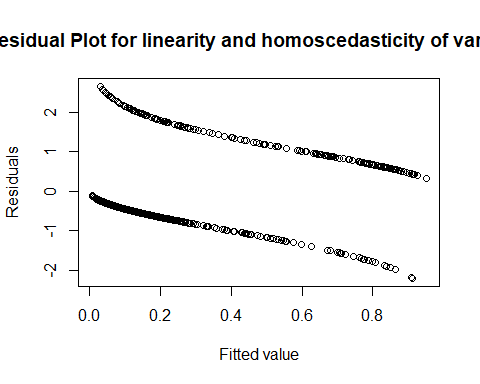


#

test for normality of the residuals

hist(resid(glm_4.1),xlab="Residuals",main="Normality of the Residuals")


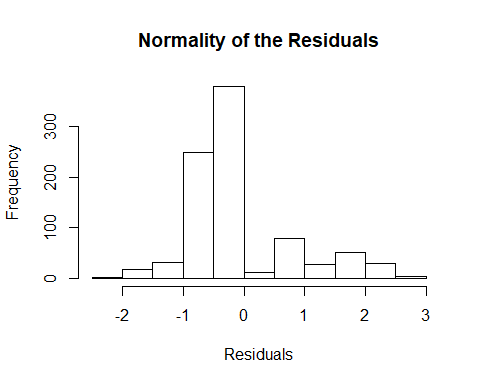


plot the residuals against each explanatory variables to test independency

plot(resid(glm_4.1)~d1_au$distance,pch=20,main="Residual Plot for independency 1",xlab="Distance (km)",ylab="Residuals")


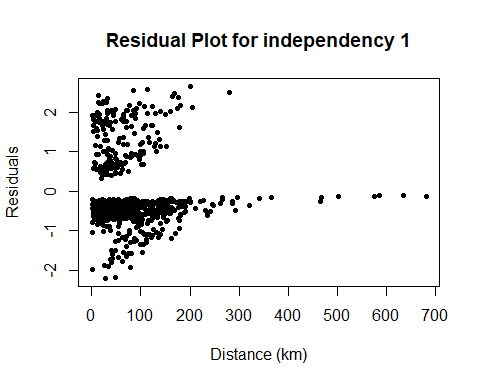


plot(resid(glm_4.1)~d1_au$effort,pch=20,main="Residual Plot for independency 2",xlab="Effort (hooks)",ylab="Residuals")


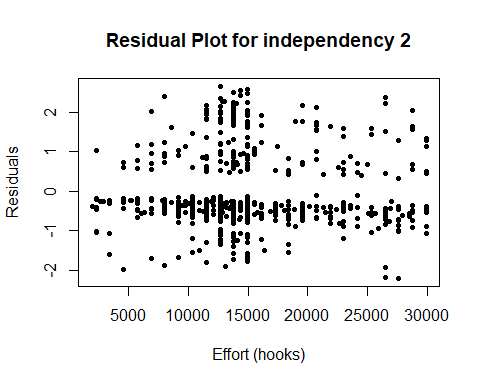


Check for temporal autocorrelation

acf(resid(glm_4.1, type="pearson"), plot=T, type="correlation")


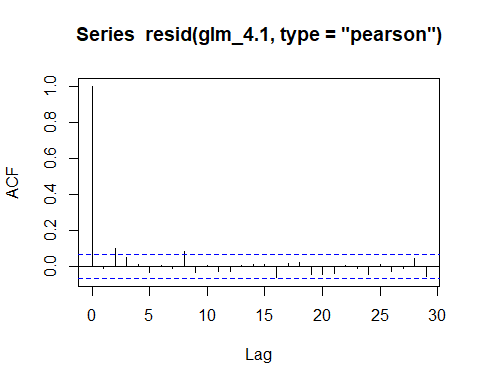

Supplement: Data S1 — Code for Generalised Linear Models fitted to the presence/absence of killer whales during fishing days [file peerj-06-5306-s001.docx]
